# Copper(II) Can Kinetically Trap Arctic and Italian Amyloid-β_40_ as Toxic Oligomers, Mimicking Cu(II) Binding to Wild-Type Amyloid-β_42_: Implications for Familial Alzheimer’s Disease

**DOI:** 10.1021/jacsau.3c00687

**Published:** 2024-02-06

**Authors:** Yao Tian, Qi Shang, Ruina Liang, John H. Viles

**Affiliations:** School of Biological and Behavioral Sciences, Queen Mary University of London, London E1 4NS, U.K.

**Keywords:** Alzheimer’s disease, amyloid, copper, kinetics, molecular mechanism

## Abstract

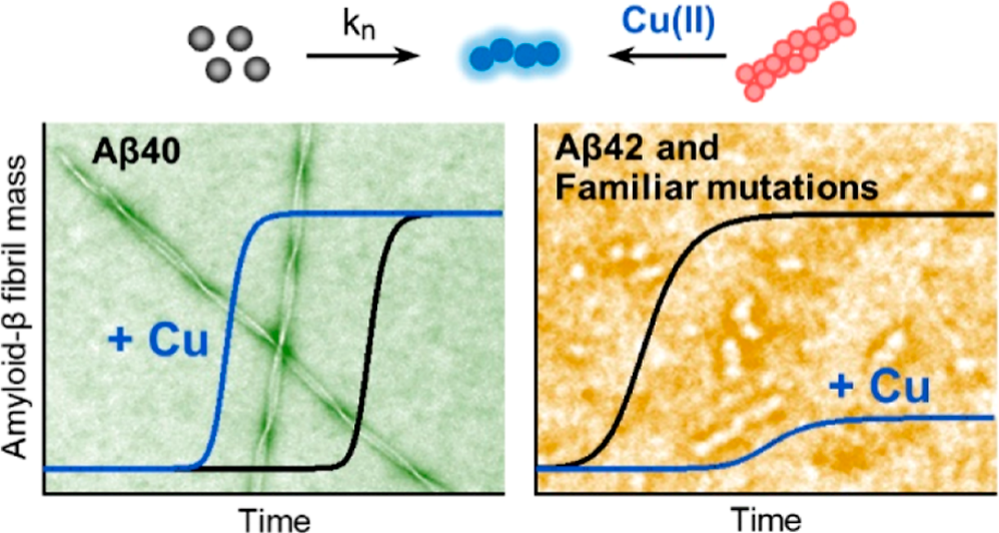

The self-association of amyloid-β (Aβ) peptide into neurotoxic oligomers is believed to be central to Alzheimer’s disease (AD). Copper is known to impact Aβ assembly, while disrupted copper homeostasis impacts phenotype in Alzheimer’s models. Here we show the presence of substoichiometric Cu(II) has very different impacts on the assembly of Aβ40 and Aβ42 isoforms. Globally fitting microscopic rate constants for fibril assembly indicates copper will accelerate fibril formation of Aβ40 by increasing primary nucleation, while seeding experiments confirm that elongation and secondary nucleation rates are unaffected by Cu(II). In marked contrast, Cu(II) traps Aβ42 as prefibrillar oligomers and curvilinear protofibrils. Remarkably, the Cu(II) addition to preformed Aβ42 fibrils causes the disassembly of fibrils back to protofibrils and oligomers. The very different behaviors of the two Aβ isoforms are centered around differences in their fibril structures, as highlighted by studies of C-terminally amidated Aβ42. Arctic and Italian familiar mutations also support a key role for fibril structure in the interplay of Cu(II) with Aβ40/42 isoforms. The Cu(II) dependent switch in behavior between nonpathogenic Aβ40 wild-type and Aβ40 Arctic or Italian mutants suggests heightened neurotoxicity may be linked to the impact of physiological Cu(II), which traps these familial mutants as oligomers and curvilinear protofibrils, which cause membrane permeability and Ca(II) cellular influx.

## Introduction

Alzheimer’s disease (AD) is responsible for the majority of dementias, and worldwide, it is estimated that 50 million people are currently suffering from this neurogenerative disease.^1^ AD pathology is dominated by the accumulation of a small peptide, amyloid-β (Aβ), that forms fibrils.^2,3^ These accumulate to form extracellular plaques, which also contain phospholipids and metal ions. Rare point mutations within Aβ such as the Arctic and Italian mutations cause early onset AD and have made a persuasive argument for the amyloid cascade hypothesis, which places Aβ self-association central to AD pathology.^4^ Aβ varies in length, with N- and C-terminal extensions and truncations, but are typically 40 and 42 amino acids long.^5^ Other genetic mutations, also associated with early onset AD, cause an increase in Aβ42 production relative to Aβ40.^6–8^ In addition, Aβ42 has a greater propensity to form fibrils and plaques, which has focused attention on Aβ42 as the toxic Aβ isoform. The small diffusible prefibrilar assemblies of Aβ42 are the most synaptotoxic.^9–14^ In contrast to fibrils, these oligomeric and extended curvilinear protofibrils are cytotoxic and can carpet the lipid membrane surface,^15^ and form ion-channel pores,^16^ which cause membrane permeability and a loss of cellular homeostasis.^17^

Potentially, metal ion binding such as Cu(II) and Zn(II) can influence Aβ synaptic toxicity by accelerating fibril formation or promoting prefibrillar toxic oligomers.^18–20^ While the redox properties of copper ions can produce reactive oxygen species causing oxidative stress observed in AD.^21,22^ Copper homeostasis is disrupted in AD patients.^23^ Levels of labile copper are found elevated, but only in the most effected regions of the AD brain,^24^ and also in blood plasma.^25^ The copper ions are concentrated in senile plaques directly bound to Aβ,^26–28^ with a fourfold increase in the levels of Cu(II) in the neuropil. Furthermore, the heightened AD phenotypes in both drosophila^29,30^ and rabbit models^31^ are linked to disrupted copper homeostasis. Additionally, an unbiased screening of 140,000 compounds has shown that copper chelators, such as clioquinol, ameliorate Aβ toxicity in a yeast model for AD.^32^ Furthermore, clioquinol can improve AD phenotypes in mice models,^33^ although this has not been replicated in human trials.^34^

Both Aβ40 and Aβ42 bind to Cu(II) with the same picomolar affinity (conditional *K*_d_ = 54 pM at pH 7.4) and 1:1 stoichiometry.^35^ Thus, in vivo Aβ (at ca. 0.5 nM at the synapse^36^) is expected to compete for Cu(II) which is thought to be released from the synapse at much higher levels, 15–250 μM, within the synaptic cleft, especially during depolarisation.^37,38^ Weaker Cu–Aβ dissociation constants have been reported, but these are determined in the presence of Cu(II) competitive buffers (such as Tris and phosphate buffer) that reduce the apparent affinity for Cu(II), when a correction is made for this, similar picomolar conditional dissociation constants are calculated.^39^

A large array of approaches have revealed that Cu(II) forms a tetragonal complex with Aβ, containing two nitrogen and two oxygen ligands in the equatorial plain.^35,40,41^ While Cu(II) binds to Aβ40 or Aβ42 with the same coordination geometry.^35^ The copper complex involves the N-terminal third of Aβ, indeed there is no difference in the Cu(II) complex formed with Aβ(1–16) compared to Aβ40 and Aβ42.^35,39,41^ The complex is dynamic and forms a number of interchangeable ligands which include two of the three histidine imidazole nitrogen’s (His6, His13, and His14) along with carboxyl coordination (e.g., Asp1, Asp7, and Glu11).^35,42–44^ Solid-state nuclear magnetic resonance (NMR) and pulsed electron paramagnetic resonance (EPR) spectroscopy of the Cu^2+^ complex indicates that the fibrillar form of Aβ40 can accommodate Cu^2+^ coordination.^45,46^

The impact Cu(II) has on Aβ40 and Aβ42 fibril assembly has been peppered with conflicting reports; both acceleration and inhibition of fibril formation have been reported. Much of the conflicting observations can be accounted for by differing experimental conditions. Furthermore, in the early studies, little distinction was made between amorphous aggregation, amyloid formation, and prefibrillar oligomer formation. It is now established, that supra-stoichiometric amounts of Cu(II) bound to Aβ tends to promote amorphous aggregates but not ordered amyloids, as the second copper ion bound causes Aβ to become insoluble.^47^ Substoichiometric levels of Cu(II) binding to Aβ are more representative of Cu(II) loading on Aβ in vivo and affect Aβ quite differently. In the case of Aβ42, most studies report substoichiometric amounts of Cu(II) inhibit fibril formation.^48–50^ Here, we will show Cu(II) actually traps Aβ as prefibral oligomers, which upon the removal of Cu(II) will nucleate (or seed) fibril formation. The impact of fibril formation with substoichiometric addition of Cu(II) to Aβ40 remains poorly established with conflicting observations. Some studies report Cu(II) to accelerate Aβ40 fibril formation,^22,47,48,51^ but this is not universally reported.^52,53^ In addition, there are now studies that have focused on Cu(II)–Aβ interactions for various point mutations, these include Iowa (D23N), A2 V, and D7H, which are linked to inherited AD.^54–56^

Like Cu(II), Zn(II) also binds to Aβ via histidines and carboxylate coordination, forming a dynamic, rapidly exchanging complex.^39,57–61^ Trace levels of Zn(II) (0.01 mol equiv) profoundly influence fibril assembly by rapidly exchanging between Aβ peptides.^62^

It is assumed that Aβ42 heightened neurotoxicity relative to Aβ40 is associated with heightened oligomer and fibril formation. Here we show a marked difference in the way Cu(II) influences assembly of nonpathogenic Aβ40 compared to pathogenic Aβ42. In particular, substoichiometric Cu(II) can trap Aβ42 in an oligomeric form but not Aβ40. We wondered if early onset AD, caused by point mutations such as Arctic and Italian Aβ40 mutations, is also influenced differently by Cu(II) compared to the wild-type Aβ40. Here we revisit copper’s impact on fibrilization for both Aβ40 and Aβ42, by globally fitting the kinetic fibril growth curves to individual microscopic rate constants, so as to understand the mechanism behind the switch in impact of Cu(II), as a promoter and inhibitor of fibril formation. With the identical copper coordination geometry for Aβ40 and Aβ42, the switch in behavior might be accounted for by the very different fibril structures, which form “U”- and “S”-shaped fibrils, for of Aβ40 and Aβ42, respectively, Figure S1. The difference in structure is caused by Coulombic interactions between the Lys28 amino group and the carboxylate at Asp23 or the C-terminal carboxylate at Ala42, Figure S1. To probe this question, we have explored a series of Aβ analogues including, the C-terminally amidated and N-terminally acetylated Aβ, together with familiar mutants, the Arctic (D22G) and Italian (D22K) for Aβ40 and Aβ42 familial isoforms. This has helped us understand how different Aβ isoforms, and their associated tendency to form different fibril structures, are impacted by Cu(II).

## Results and Discussion

### Contrasting Influence of Cu(II) on the Kinetics of Fibril Assembly for Aβ40 and Aβ42

**Figure 1:**
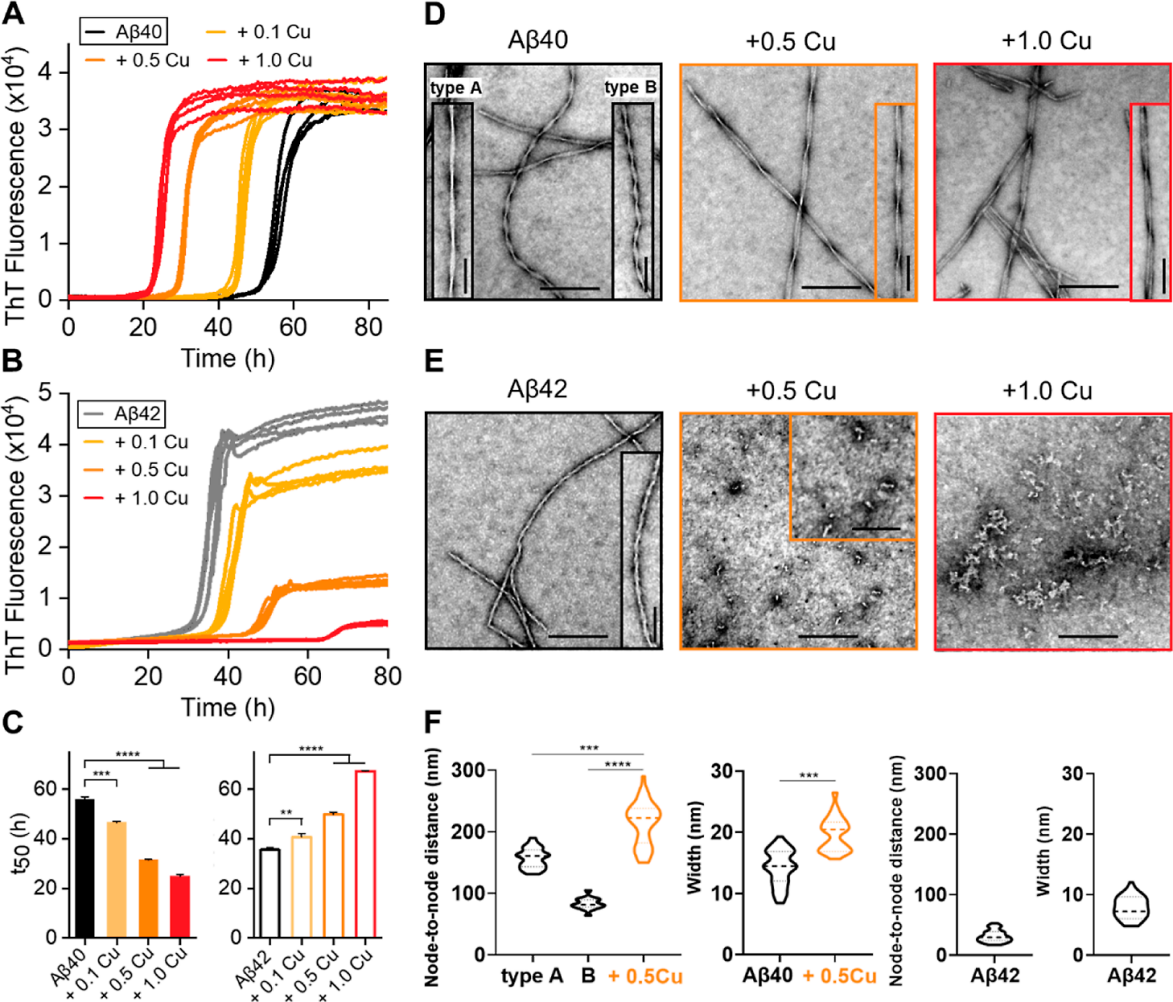
Effect of Cu^2+^ on Aβ40 and Aβ42 fibril kinetics profiles. Aβ40 (A) and Aβ42 (B) both 10 μM in the absence and presence of 0.1, 0.5, and 1.0 mol equiv of Cu^2+^, from black line to red line, respectively. (C) Change in *t*_50_ versus Cu^2+^, error bars are standard error of the mean (SEM) from four replicates. One-way ANOVA test, **P ≤ 0.01, ***P ≤ 0.001, and ****P ≤ 0.0001. Negatively stained TEM fibril images produced at 0, 0.5, and 1.0 mol equiv of Cu^2+^ for Aβ40 (D) and Aβ42 (E). Scale bars: 200 nm; inset 100 nm. (F) Node-to-node distance of Aβ40 and Aβ42 fibril twists and fibril width in the absence and presence of 0.5 mol equiv of Cu^2+^. *N* = 50 individual fibrils are measured per condition. Long and short twists are designated as types A and B, respectively. Preparations were incubated with 20 μM ThT in 30 mM HEPES and 160 mM NaCl buffer (pH 7.4) at 30 °C quiescently.

First, we wanted to determine the influence of substoichiometric Cu(II) on the kinetics and structure of both wild-type Aβ40 and Aβ42 fibril formation. Although this has been the subject of interest for several years, the influence of Cu(II) on Aβ40/42 assembly still remains disputed.^22,47–53^Figure 1A shows the kinetics of Aβ40 as monitored by the amyloid specific dye thioflavin T (ThT). Increasing levels of Cu(II) causes accelerated fibril formation kinetics with a significant shortening of lag-times from a mean of 56.0 ± 0.9 h for Aβ40 in the absence of Cu(II) to 24.9 ± 0.8 h for Cu–Aβ40 at 1:1 stoichiometric loading, as summarized in Figure 1C and Table S1.

It has been suggested that Cu(II) might interfere with the ability of ThT to detect fibrils by quenching the ThT fluorescence; however, we observe no evidence of this. The ThT kinetic traces, shown in Figure 1A, are for absolute ThT intensity, and are not normalized. Cu(II) does not impact the ability to detect fibrils of Aβ40, and there is no change in the total ThT signal, Figures 1A, S2A, and Table S1. This is supported by TEM images, Figures 1D and S3, which show a similar abundance of fibrils for both Cu(II)-loaded and Cu(II)-free Aβ40.

Next, we performed a similar ThT-monitored fibril assembly experiment on the more neurotoxic Aβ42, Figure 1B. Cu(II) has a starkly different impact on the fibril assembly for Aβ42; rather than acceleration of fibril assembly, the amount and rate of fibril formation are greatly reduced. Cu(II) at just 0.5 mol equiv reduces the ThT signal by more than two-thirds, Figure S2B, while Cu(II) at 1:1 reduces the ThT signal intensity even more to just 11%, while the rate of fibril formation is extended with *t*_50_ going from 35.6 ± 1.1 to 67.3 ± 0.3 h, see Figure 1C and Table S1. The marked reduction in the total number of fibrils indicated by the ThT signal is supported by TEM images that show almost no fibrils present for images of Aβ42 incubated with 0.5 mol equiv of Cu(II), see Figures 1E and S4. Quantification of the total fibril load from the TEM micrographs match the marked drop in ThT signal with an 84% reduction in of the number of fibrils present in the Cu(II) loaded sample (*n* = 50 images inspected). Instead, many prefibril assemblies are observed; short oligomers and more extended curvilinear protofibrils, but almost no fibrils, Figures 1E and S4.

We wondered if the conflicting behavior reported indicating Cu(II) inhibition of Aβ40 fibril formation rather than acceleration,^52,53^ was due to differences in experimental conditions, particularly the use of phosphate buffer, which can form insoluble Cu(II)–phosphate. However, the presence of phosphate buffer, in our study, did not alter the impact of Cu(II) on Aβ40 assembly, and like the data in Figure 1A, Aβ40 fibril formation is accelerated by Cu(II) addition, up to 1 mol equiv, see Figure S5. Furthermore, the acceleration of Aβ40 by Cu(II) has been reported by others,^22,47,48,51^ thus the difference in the reported influence of Cu(II) on Aβ40 fibril formation kinetics is quite surprising and is difficult to account for.

### Different Impact of Cu(II) on the Structure of Aβ40 and Aβ42 Assemblies

Amyloid fibrils are well-known for their ability to form polymorphic structures.^63,64^ The TEM images suggest copper-free Aβ40 has two distinct fibril morphologies, even when generated from size exclusion chromatography (SEC) purified Aβ40 monomer. The node-to-node periodicity in the twist are 157 ± 16 nm (designated type-A), and 83 ± 1 nm (type-B) shown in Figure 1F. When Aβ40 is incubated with Cu(II), copper binding directs the type of fibrils produced into a single morphology type, similar to type-A with the longer twist of 242 ± 50 nm. The fibril morphology is typically determined by the way protofibrils packed together to form a twisting fibril.^63,64^ This suggests the presence of Cu(II) restricts the type of protofibril packing possible; this mechanism is illustrated in Figure S6.

**Figure 2:**
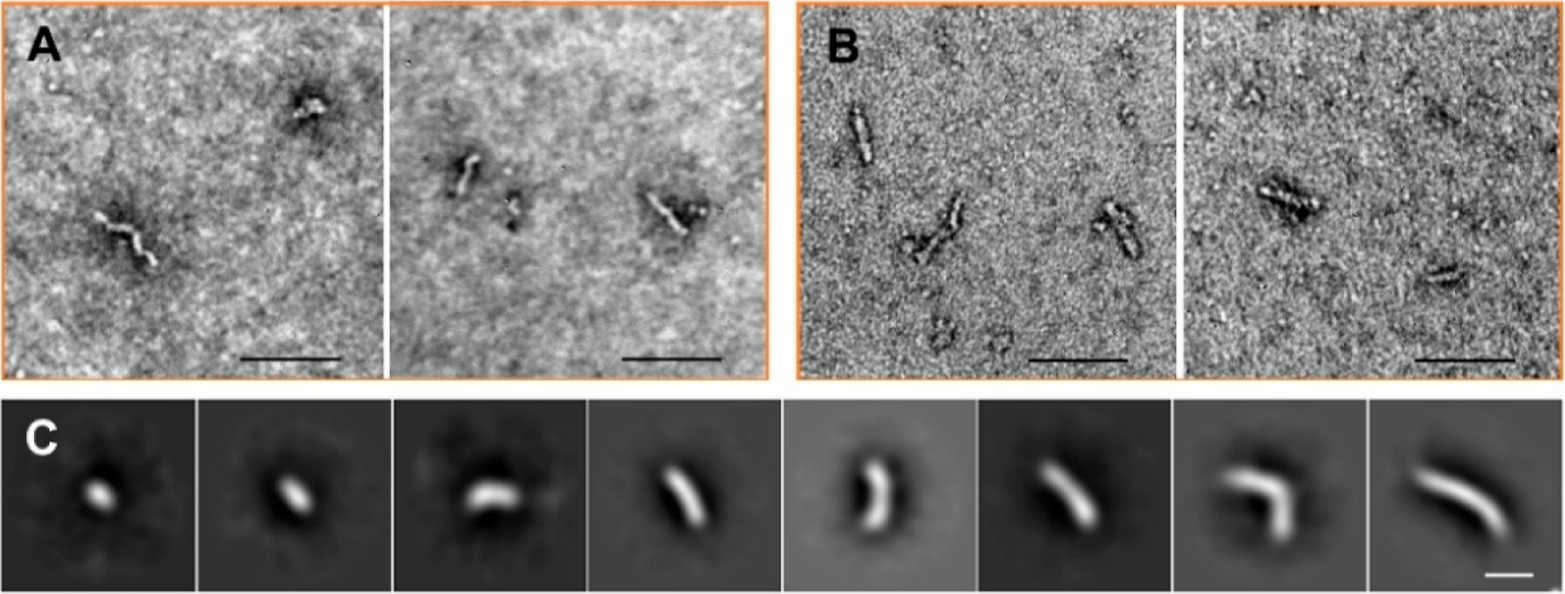
TEM images of Cu^2+^-trapped Aβ42 oligomers. (A) TEM images of 10 μM Aβ42 incubated with 5 μM Cu(II). (B) TEM images of oligomers from preformed fibrils with subsequent addition of Cu(II), scale bars: 200 nm. (C) 2D class averages of oligomers and curvilinear protofibrils for Aβ42 trapped by Cu(II), ca. 100 single particles are shown for each 2D class average, scale bar 5 nm.

As already shown in Figure 1E, almost no fibrils of Aβ42 are observed when Cu(II) is incubated with Aβ42; however, there are many prefibrillar assemblies observed; further images are shown in Figures 2 and S4. Small oligomers and extended oligomers, known as curvilinear protofibrils, are widespread. These assemblies have been subject to single particle analysis of the negatively stained images, typical 2D class-averages are shown in Figure 2C. These prefibrillar assemblies are indistinguishable from the oligomers and curvilinear protofibrils but only transiently observed during copper free assembly at the end of the lag-time.

It is well established that oligomers and the curvilinear protofibrils are the cytotoxic form of Aβ, rather than fibrils.^11,14,65,66^ The influence of the Cu(II) promoted oligomers on membrane permeability, Ca(II) cellular influx, and cytotoxicity is described later.

### Microrate Constants, Copper’s Impact on Primary Nucleation

**Figure 3:**
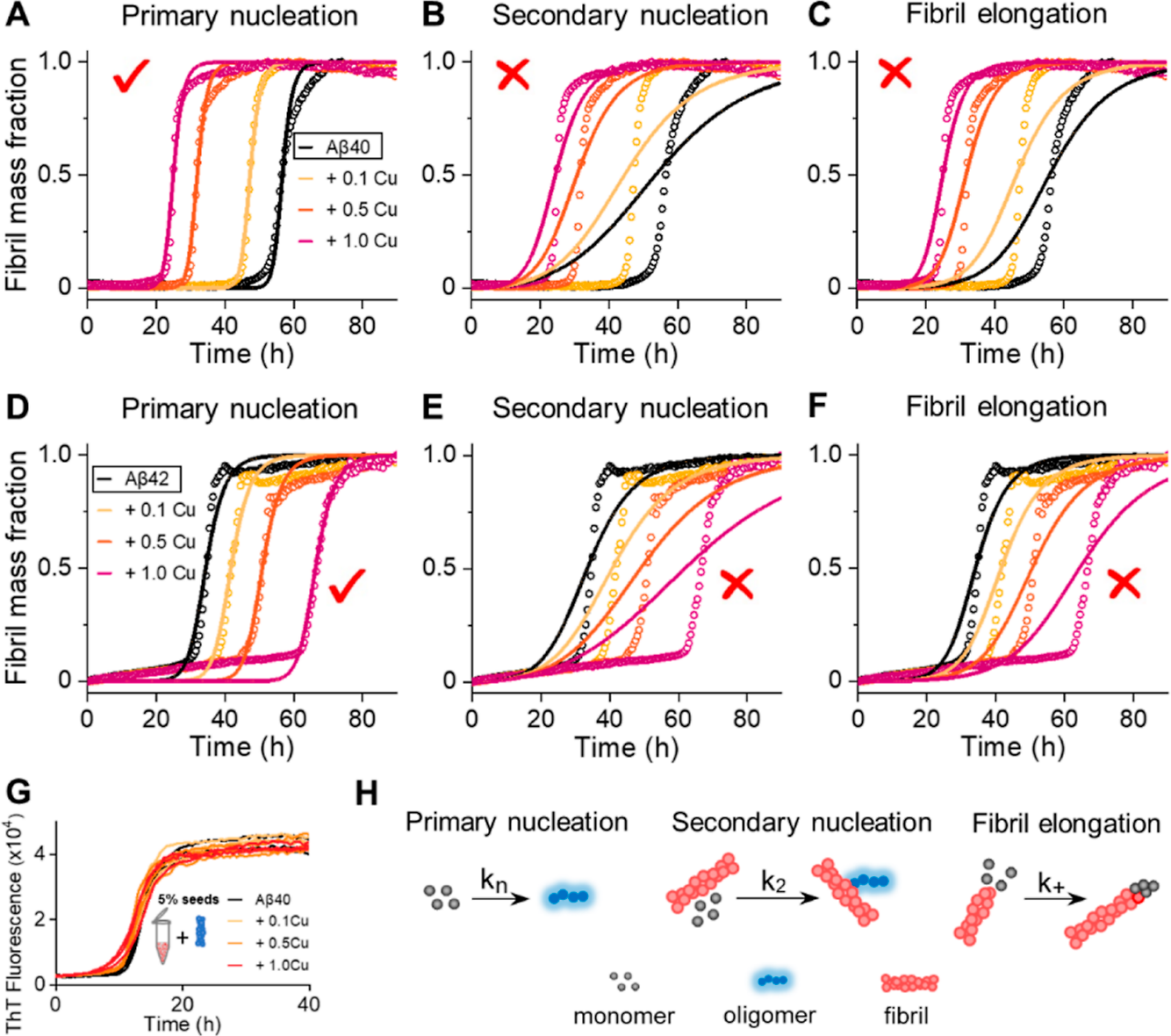
Cu^2+^ effects the primary nucleation process of Aβ40 and Aβ42 aggregation. Normalized kinetic profiles of 10 μM Aβ40 (A–C) and Aβ42 (D,E) in the absence and presence of 0.1, 0.5, and 1.0 mol equiv of Cu^2+^, from black line to red line, respectively. The solid lines represent global fits of the kinetic traces when only primary nucleation (*k*_n_) (A,D), secondary nucleation (*k*_2_) (B,E) and fibril elongation (*k*_+_) (C,F) rate constants are altered. (G) Aβ40 with 5% fibril seeds in the absence and presence of 0.1, 0.5, and 1.0 mol equiv of Cu^2+^. (H) Schemes of the microscopic steps for primary nucleation, secondary nucleation, and fibril elongation.

To understand the mechanism of this acceleration and retardation of Aβ40 and Aβ42 fibril formation, we have globally fitted the ThT kinetic data to a set of analytical rate equations.^67^ This has enabled us to determine in what way Cu(II) impacts the individual microscopic molecular processes of assembly. The macroscopic kinetic curves are described in terms of primary nucleation (*k*_n_), secondary nucleation (*k*_2_), and elongation rates (*k*_+_), using the online fitting program, AmyloFit.^67^Figure 3A shows a good fit to the kinetic data in which only primary nucleation (*k*_n_) is permitted to vary with increasing Cu(II) addition to Aβ40, while *k*_2_ and *k*_+_ are fixed at a single value by globally fitting these rate constants. In contrast, if *k*_n_ is fitted to a single value and the secondary nucleation or elongation rate constants are permitted to vary, a fit to the set of macroscopic fibril formation curves is not achieved, Figure 3B,C. These globally fitted traces indicate Cu(II) accelerates Aβ40 fibril formation only via a change in primary nucleation (*k*_n_). To support this assertion, we generated ThT kinetic traces seeded with 5% fibrils; this has the effect of circumventing primary nucleation (*k*_n_). These seeded fibril growth curves are unaffected by the presence of Cu(II), Figure 3G. This confirms that copper’s impact of Aβ40 fibril assembly is dominated by changes in primary nucleation and has little impact on *k*_2_ and *k*_+_.

The acceleration in fibril formation via primary nucleation can be explained by Cu(II) adding a positive charge to Aβ. At physiological pH 7.4, Aβ’s histidine residues are largely deprotonated; thus, the binding of copper adds two positive charges. Aβ has a pI of 5.3; therefore, Cu(II) binding makes Aβ more neutrally charged and more prone to self-association and fibril formation. We have recently reported a similar acceleration of primary nucleation as positive charge, in the form of protons, is added to Aβ by lowering the pH. Like Cu(II) addition, the change in pH also only accelerates primary nucleation.^68^

Fibril growth curves for Aβ42 have also been fitted to individual microscopic rate constants in the presence of increasing Cu(II). Cu(II) has a very different effect on Aβ42 assembly compared to Aβ40. The reduction in fibril growth rates with Cu(II) addition appears to be caused by a decrease in primary nucleation. The reduction in the rate of fibril formation and the loss of total fibril mass could be due to the removal of available Aβ42 monomers capable of forming fibrils; however, the change in lag-time could also be caused by a change in the rate of fibril dis-assembly. The following section makes it clear that Cu(II) can dissociate preformed Aβ42 fibrils. The molecular process associated with Cu(II)-mediated Aβ42 assembly/disassembly is discussed further in the following section.

### Cu(II)-Induced Disassembly of Aβ42 Fibrils: EDTA Chelator and Cu(II) Addition to Preformed Fibrils

**Figure 4:**
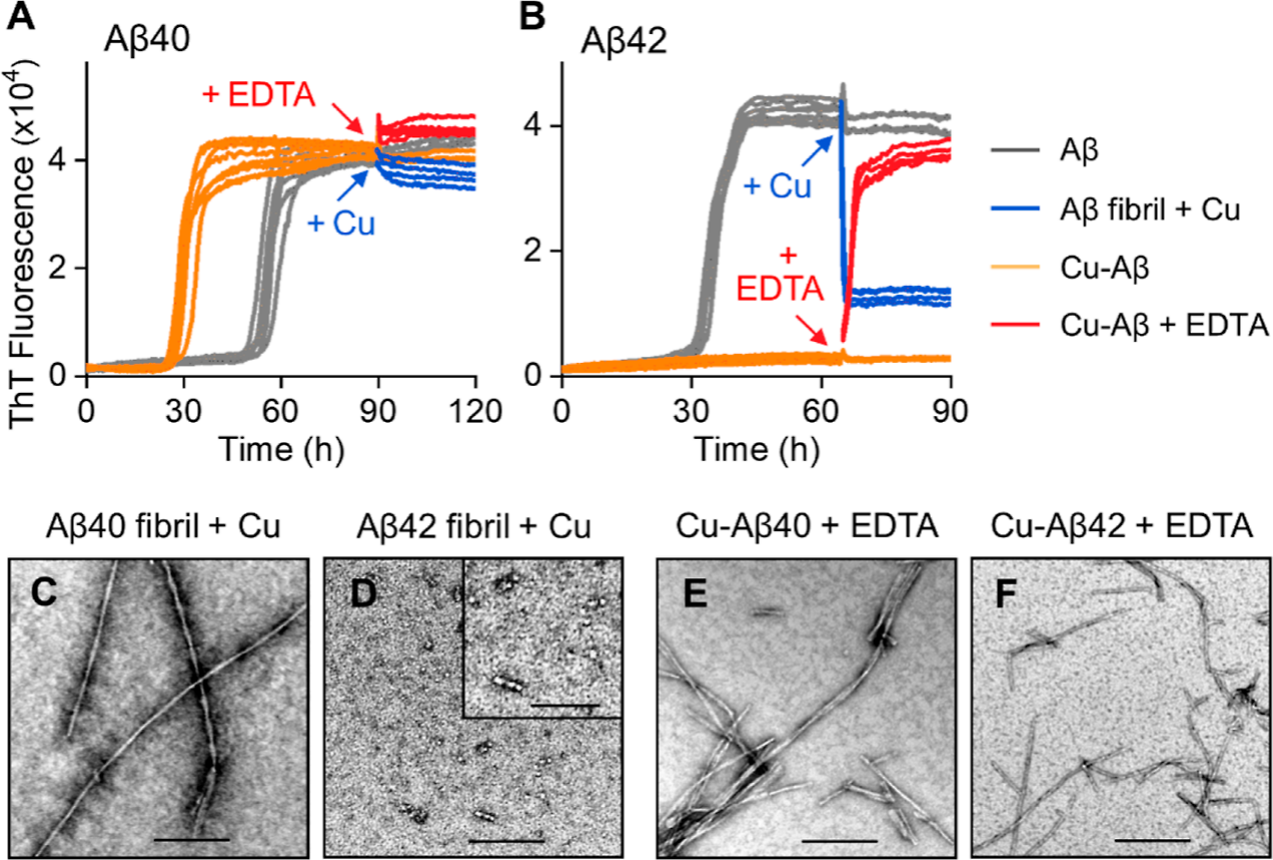
Switching on/off the fibril growth of Aβ by EDTA and Cu^2+^. Kinetics profiles of 10 μM Aβ40 (A) and Aβ42 (B) in the absence (gray) and presence (orange) of 8 μM Cu^2+^. 8 μM Cu^2+^ (8 μM; blue) or 50 μM EDTA (red) was added to half of the samples at 90 h for Aβ40 and 65 h for Aβ42. *N* = 4 traces for each condition. TEM images of Aβ40 (C) and Aβ42 (D) produced with Cu^2+^ added to the preformed fibrils. Aβ40 (E) and Aβ42 (F) in the presence of Cu^2+^ with subsequent EDTA addition. Scale bars: 200 nm; inset 100 nm.

To further probe the Cu(II)-dependent effects on fibril assembly, we used a tight Cu(II) chelator, EDTA, to remove Cu(II) from Aβ, Figure 4. While in the reverse experiment Cu(II) has been added to preformed fibrils, Figure 4. As expected, from Figure 1A, the addition of Cu(II) to preformed Aβ40 fibrils had no significant effect on the ThT fibril signal. Similarly, addition of EDTA to copper loaded Aβ40 fibrils had no impact on the total amount of fibrils. This also confirms that Cu(II) does not quench the ThT signal. The effect EDTA has on Cu–Aβ42 is very different. Upon the addition of EDTA, the ThT fluorescence signal for fibrils rapidly increases and reaches a ThT intensity comparable to that of Aβ42 in the absence of Cu(II). There are two important observations to note about this behavior. The inhibitory effect of Cu(II) on Aβ42 fibril formation is completely reversible with the removal of Cu(II) by EDTA. Also, upon the removal of Cu(II), Aβ42 fibrils form very rapidly. The lag-time to fibril formation is reduced from 32 h to just 0.8 h, and the *t*_50_ is just 3.2 h after addition of EDTA. This indicates the Cu(II)-trapped, oligomers and curvilinear protofibrils are able to, nucleate (seed) fibril formation. The growth time (slope) is similar for the seeded fibril formation; this indicates the seeds impact primary nucleation (the lag-time), rather than secondary nucleation and elongation (the growth-time), which require fibrils to be present as well as monomer.

**Figure 5:**
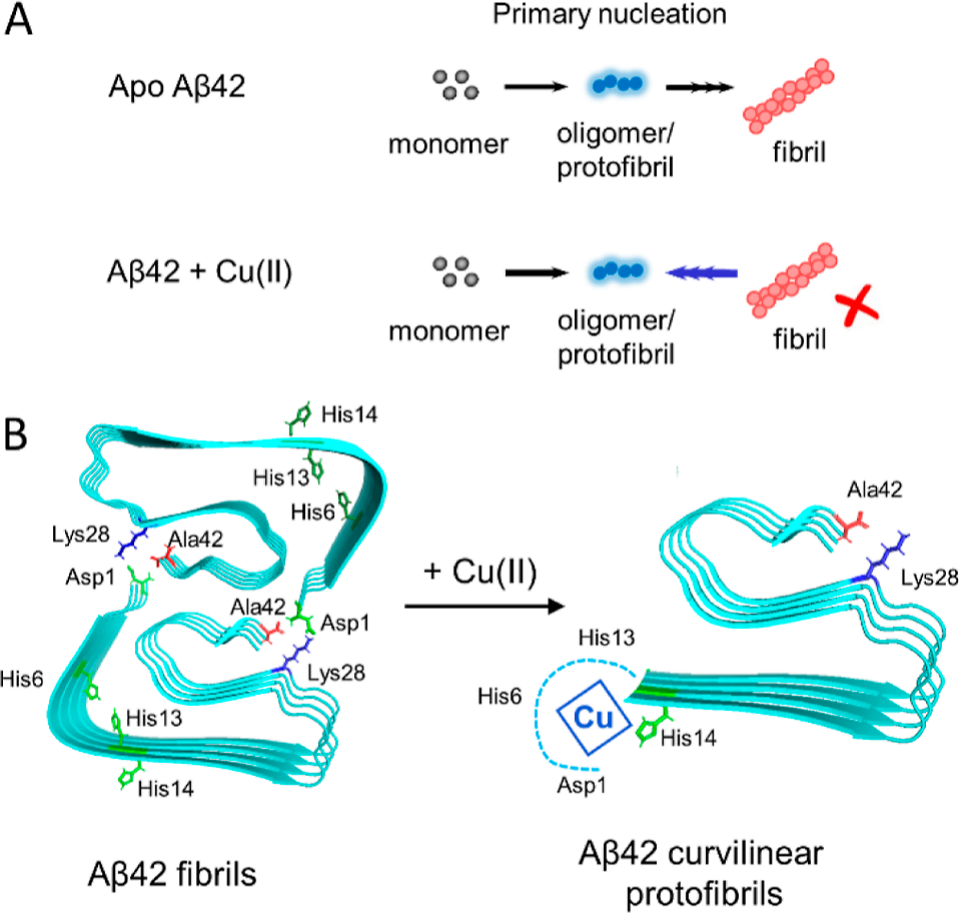
Scheme of Cu(II)’s impact on Aβ42 fibrilization. (A) Primary nucleation; the kinetic steps to go from monomer to fibrils, in which oligomer formation is the rate-limiting step. Addition of Cu(II) causes Aβ42 to dissociate from the fibril to form protofibrils. (B) Aβ42 fibrils are formed from the packing of two protofibrils. Key residues in the N-terminus bind Cu(II) and disrupt the electrostatic packing of protofibrils, causing the fibrils to dissociate. Fibril structure from pdb 5OQV.^71^

Most surprising is the marked effect upon the addition of Cu(II) to preformed Aβ42 fibrils. Despite the well documented stability of fibrils, the addition of Cu(II) to Aβ42, but not Aβ40 fibrils, causes rapid disassembly of amyloid fibrils, Figure 4. The effect has incorrectly been interpreted as Cu(II) interfering with the detection of fibrils by ThT, however, the complete loss of fibrils, imaged by TEM, confirms Cu(II) efficiently disaggregates amyloid fibrils of Aβ42 (but not Aβ40), Figure 4C–E. The observation that Cu(II) is able to disassemble preformed Aβ42 fibrils indicates that the mechanism of inhibition is not removing an available pool of Aβ42 monomer; the action of Cu(II) involves the reversal of fibril assembly. Cu(II) does not just slow fibril formation but to a large extent can reverse and disassemble preformed fibrils. These observations (Figure 4) together with the microscopic kinetic analysis (Figure 3) can lead us to a scheme of fibril assembly behavior in the presence of Cu(II), as shown in Figure 5. We suggest that Cu(II) disrupts the packing between protofibrils and disassembles fibrils back to curvilinear protofibrils. The stability of fibrils due to hydrogen-bonding in a cross-β structure is well documented, while the action of Cu(II) is between protofibrils, which are only formed from electrostatic and hydrophobic interactions and not via regular hydrogen-bonding, see Figure 5.

### Cu(II) Promoted Aβ Cytotoxicity and Cellular Membrane Permeability

**Figure 6:**
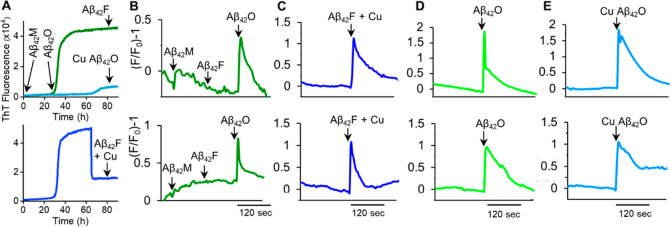
Calcium influx of HEK293T cells in response to different Aβ42 preparations. (A) ThT fibril growth curves indicating the five Aβ42 preparations used. HEK293T cells are loaded with Ca(II) sensitive fluorescent dye, Fluoro3-AM. (B) Time-lapse recording of fluorescence relative to fluorescence before addition of Aβ42 monomer(Aβ_42_M), and subsequent addition of Aβ42 fibril (Aβ_42_F), Aβ42 oligomer (Aβ_42_O). Notably, only Aβ42 oligomers cause calcium influx. (C) Aβ42 fibril disassembled into oligomers, by Cu(II) addition, causes Ca(II) influx in HEK cells. (D) Aβ42 oligomers from the lag-phase causes Ca(II) influx. (E) Aβ42 assembly trapped as oligomers by Cu(II) and causes Ca(II) influx. Aβ42 (5 μM) is added to extracellular media, buffered at pH 7.4.

Aβ cytotoxicity is believed to be caused by membrane permeability, this results in unregulated Ca(II) cellular influx into the lumen.^9–14,17,43^ The Ca(II)-sensitive fluorescent dye Fluoro3-AM has therefore been used to monitor cellular membrane permeability in the presence of different forms of Aβ42, Figure 6. Figure 6A highlights five different Aβ42 preparations used from various stages of fibril formation. Chromatographic purified monomeric Aβ42, added to the extracellular medium (5 μM) has no effect on Ca(II) levels within the cell lumen, Figure 6B. Similarly Aβ42 fibrils, taken at the plateau phase of fibril growth, have no impact on cellular Ca(II) levels, also shown in Figure 6B. While in contrast, oligomers of Aβ42, taken at the end of the lag-phase, cause considerable Ca(II) influx, within a minute after exposure to the cell, Figure 6B. Importantly, oligomeric preparations, produced by disassembly of Aβ42 fibrils induced by Cu(II) addition, are also capable of causing considerable membrane permeability and Ca(II) cellular influx, Figure 6C. In addition, a preparation in which Cu(II) traps Aβ42 as oligomers (preparations that would otherwise have formed fibrils), also cause profound membrane permeability, Figure 6E.

The ability for Cu(II) to trap Aβ42 into toxic oligomeric assemblies or convert Aβ42 fibrils into toxic oligomers has a profound implication for copper’s impact on Aβ42 cytotoxicity. In vivo, heightened neurotoxicity of Aβ42, relative to Aβ40, could conceivably be due to the difference in the way physiological Cu(II) impacts Aβ assembly; trapping only Aβ42 as toxic oligomers.

### Differential Effects of Cu(II) on Aβ40 and Aβ42 Fibril Formation, Probed with a C-Terminal Amidated Analogue

We were intrigued by the very different impact Cu(II) has on the assembly and disassembly of amyloid fibrils for Aβ40 compared to pathogenic Aβ42. It is very well established that Cu(II) binds to these isoforms with the same coordination geometry and affinity; forming an interchangeable (2N+2O ligands) tetragonal complex with two of the three histidines (His6, His13, or His14) and carboxylates (e.g., Asp1, Asp7, or Glu11).^35,40–44^ This suggests that the differences must be associated with the structure of fibrils formed, rather than a difference in Cu(II) coordination. There are now several structures reported for Aβ40 and Aβ42 determined by cryoEM and solid-state NMR.^69–74^

An interesting consequence of the addition of two amino acids at the C-terminus of Aβ42 is the ability for the C-terminal carboxylate to form Coulombic interactions with the amino group of Lys28.^69,70^ This interaction is not typically favored for the C-terminus of Aβ40 fibrils; instead, the salt-bridge occurs between Lys28 and Asp23, see Figure S1. A consequence, of the “S”-shaped arrangement, reported in both recombinant fibrils and brain derived Aβ42 fibril structures^71,74^ is a role for the N-terminal residues which typically form part of the protofibril interface, see Figure 5. While for Aβ40 fibrils the Cu(II) binding N-terminal residues remain unstructured in the fibrils,^70^ thus the N-terminal residues seem less necessary for the protofibril interface, Figure S6.

**Figure 7:**
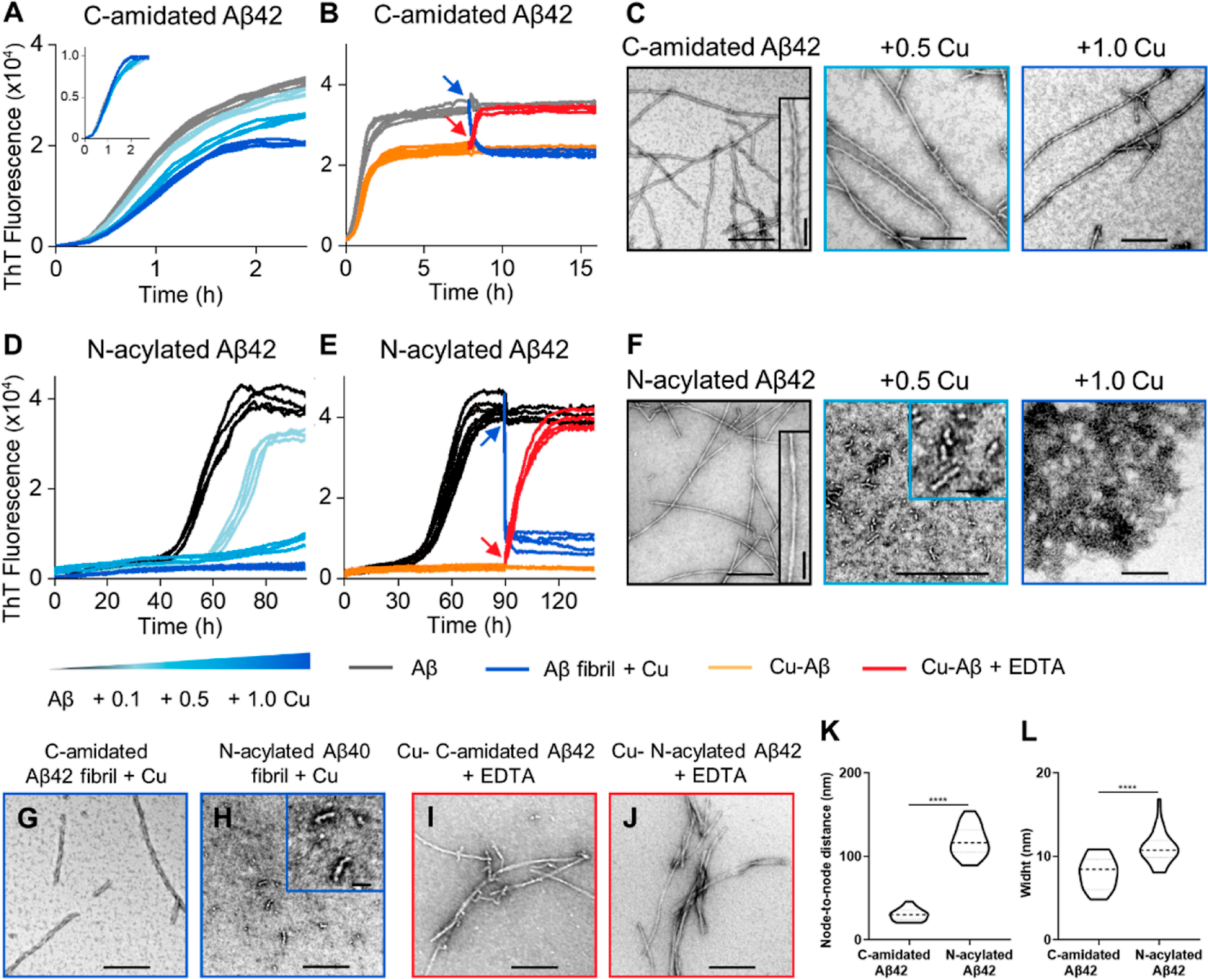
Cu(II) impact on C-amidated Aβ42 and N-acylated Aβ42 aggregation. Cu(II) has relatively little impact on C-amidated Aβ42 fibril formation, in contrast to wild-type Aβ42 and N-terminal amidated Aβ42, which are trapped as oligomers by Cu(II). Kinetics profiles of 10 μM C-amidated Aβ42 (A) and N-acylated Aβ42 (D) in the absence and presence of 0.1, 0.5, and 1.0 mol equiv of Cu^2+^. Inset shows normalized ThT fluorescence. Kinetics profiles of 10 μM C-amidated Aβ42 (B) and N-acylated Aβ42 (E) in the absence (black/gray) and presence (orange) of 8 μM Cu^2+^. 8 μM Cu^2+^ (8 μM; blue) or 50 μM EDTA (red) was added to half of the samples at 8 h for C-amidated Aβ42 and 90 h for N-acylated Aβ42. *N* = 4 traces for each condition. TEM fibril images produced at 0, 0.5, and 1.0 mol equiv of Cu^2+^ for C-amidated Aβ42 (C) and N-acylated Aβ42 (F). TEM images of C-amidated Aβ42 (G) and N-acylated Aβ42 (H) produced with Cu^2+^ added to preformed fibrils. C-amidated Aβ42 (I) and N-acylated Aβ42 (J) were analyzed in the presence of Cu^2+^ with subsequent EDTA addition. Scale bars: 200 nm; inset 30 nm. (K) Node-to-node distance of C-amidated Aβ42 and N-acylated Aβ42 fibril twists. (L) Fibril widths. *N* = 50 individual fibrils are measured per condition.

To test the importance of the C-terminal carboxylate in forming a Coulombic interaction with Lys28, we studied a C-terminally amidated analogue of Aβ42, which is unable to form a Coulombic interaction with the Lys28 amino group. The C-terminally blocked analogue switches the impact of Cu(II) on Aβ42 assembly, in contrast to wild-type Aβ42, Cu(II) does not trap Aβ42_C-blocked_ as oligomers, as indicated by the strong ThT signal produces as fibrils form, Figure 7A. This is apparent from the ThT kinetic curves, Figure 7A and TEM images, Figure 7C and further images Figure S7. The effect of Cu(II) addition to performed fibrils and the impact of EDTA on the Cu-loaded fibrils has also been studied and confirms Cu(II) does not disassemble Aβ42_C-blocked_ fibrils, Figure 7B and TEM images (Figures 7G and S7) as compared to the behavior wild-type Aβ42 shown in Figure 4. This strongly supports the assertion that the different behavior of Aβ40 compared to Aβ42, are indeed produced by the difference in the fundamental fold of the fibril structures, which is caused by the presence of Ala42 carboxylate salt-bridge. Support for this switch in fibril structure for the Aβ42_C-blocked_ analogue, is derived from cross-seeding experiments,^75^ which indicate the two fibril structures are sufficiently different to be incompatible and do not cross-seed during fibril formation.

The impact on the N-terminal acetylated analogue for Aβ42 was also studied, Figures 7D–F and S8. In this case, there is no difference in the fibril assembly compared to wild-type Aβ42. This indicates that acetylation of the N-terminal amino group does not disrupt the manner in which Cu(II) traps the Aβ42 assembly as oligomers.

### Cu(II) and Familial Aβ Mutants—Arctic and Italian

There are several rare point mutations within Aβ that cluster at residues 22 or 23 and cause early onset familiar AD.^76,77^ We wondered if the mutated forms of Aβ might be influenced by Cu(II) differently from wild-type Aβ. These mutations are believed to affect the type of fibril fold, due to the loss of a Coulombic interaction between Asp23 and Lys28, observed for wild-type Aβ40.^78^

**Figure 8:**
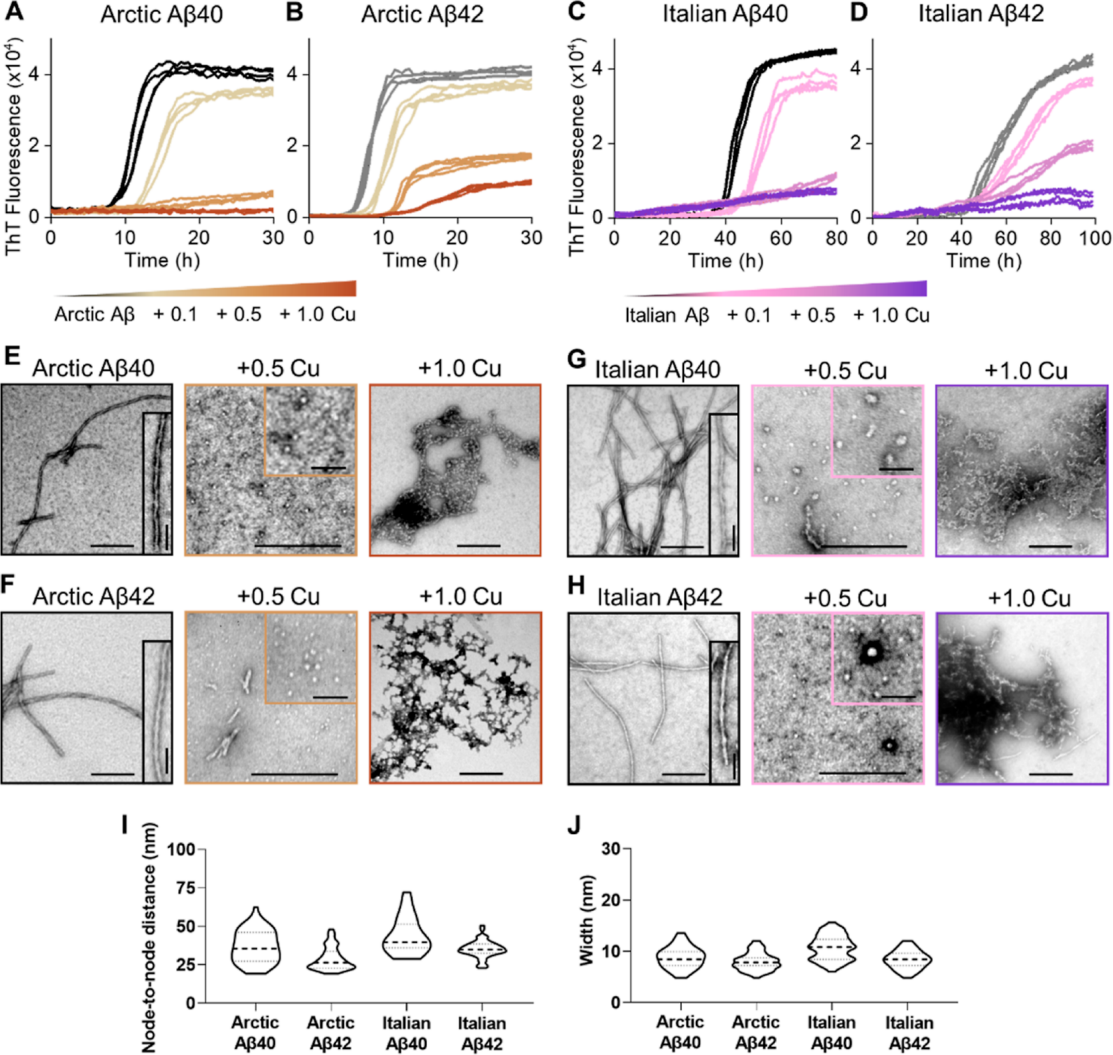
Cu^2+^ inhibits the formation of Arctic and Italian Aβ40 and Aβ42 fibril formation. Kinetics profiles of 10 μM Arctic Aβ40 (A), Arctic Aβ42 (B), Italian Aβ40 (C) and Italian Aβ42 (D) in the absence and presence of 0.1, 0.5, and 1.0 mol equiv of Cu^2+^. Negatively stained TEM fibril images produced at 0, 0.5, and 1.0 mol equiv of Cu^2+^ for Arctic Aβ40 (E), Arctic Aβ42 (F), Italian Aβ40 (G) and Italian Aβ42 (H). Scale bars: 200 nm; inset, 50 nm. (I) Node-to-node distance of fibril twists. (J) Fibril width. *N* = 50 individual fibrils are measured per condition.

**Figure 9:**
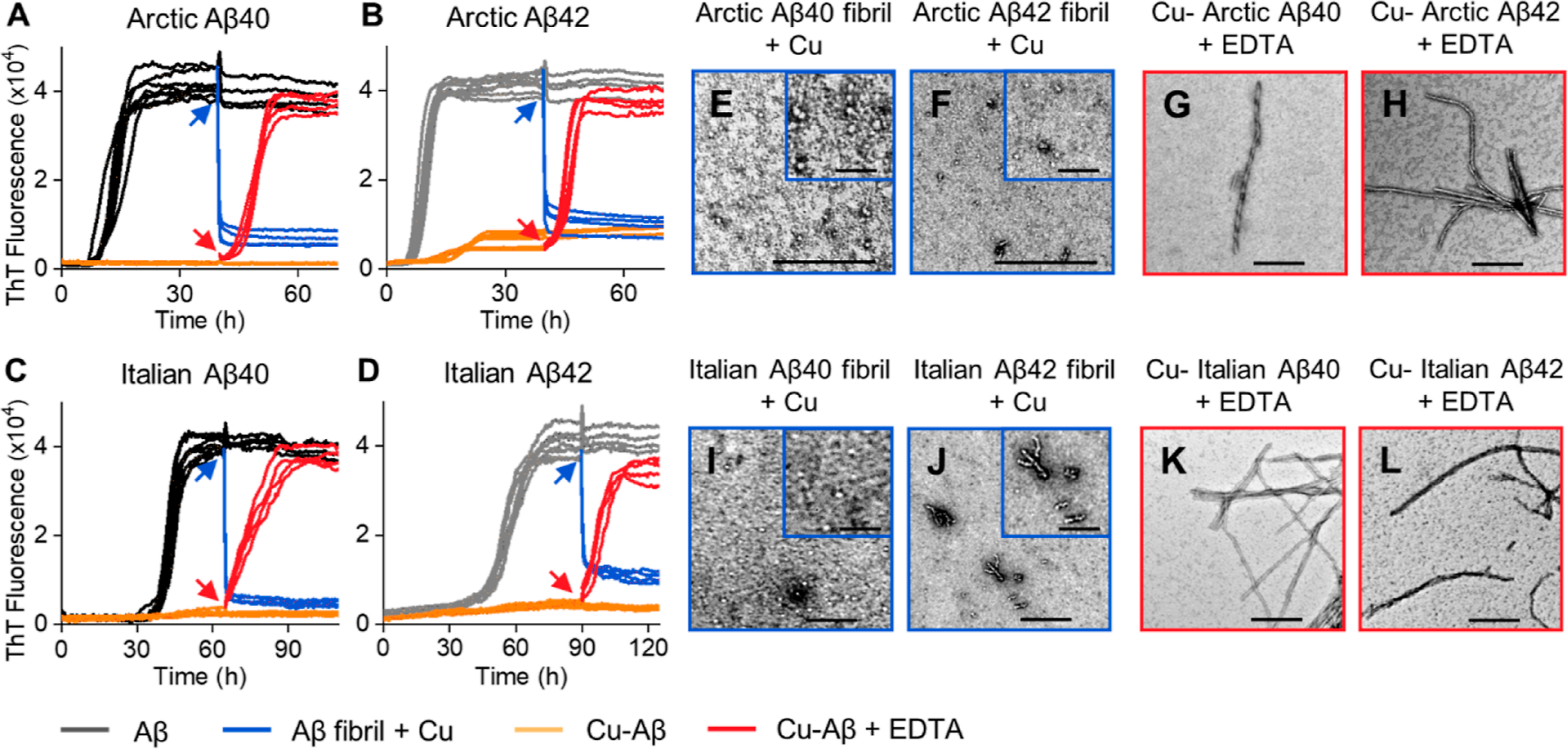
Switching on/off the fibril growth of Arctic and Italian Aβ by EDTA and Cu^2+^. Kinetics profiles of 10 μM Arctic Aβ40 (A), Arctic Aβ42 (B), Italian Aβ40 (C), and Italian Aβ42 (D) in the absence (black/gray) and presence (orange) of 8 μM Cu^2+^. 8 μM Cu^2+^ (blue) or 50 μM EDTA (red) was added to half the samples at 40 h for Arctic Aβ40/42, 65 h for Italian Aβ40 and 90 h for Italian Aβ42. Preparations were incubated with 20 μM ThT in 30 mM HEPES and 160 mM NaCl buffer (pH 7.4) at 30 °C quiescently. *N* = 4 traces for each condition. TEM images of Arctic Aβ40 (E), Arctic Aβ42 (F), Italian Aβ40 (I) and Italian Aβ42 (J) produced with Cu^2+^ added to preformed fibrils. Arctic Aβ40 (I), Arctic Aβ42 (J), Italian Aβ40 (K) and Italian Aβ42 (L) in the presence of Cu^2+^ with subsequent EDTA addition. Scale bars: 200 nm; inset 50 nm.

We have monitored fibril growth of the Arctic (E22G) and Italian (E22K) mutants, in the presence and absence of Cu(II) ions, for Aβ40 and Aβ42, Figure 8. Arctic Aβ40 and Italian Aβ40 behave very differently from wild-type Aβ40. Rather than accelerate Aβ40 fibril formation (Figure 2A), Cu(II) has an impact on fibril formation similar to wild-type Aβ42. Both the ThT signal and TEM images show marked inhibition of fibril formation with substoichiometric Cu(II) addition, Figure 8. Cu(II) traps both Aβ40 and Aβ42 for both familial mutants as oligomers. Further TEM images showing Cu(II) trapping both Aβ40 and Aβ42 mutants as protofibrils for all four isoforms are shown in Figures S9–S12. In support of this switch in behavior for the Aβ40 mutants, we performed EDTA additions and Cu(II) addition to preformed fibrils, shown in Figure 9. Again, the behavior of Aβ40 (Arctic) and Aβ40 (Italian) mimics the behavior of wild-type Aβ42 in the presence of Cu(II). This data, Figure 9, confirm Cu(II) traps Aβ40 and Aβ42 Arctic and Italian as oligomers; these oligomers are capable of rapidly seeding fibril formation upon the removal of Cu(II) with EDTA.

The Glu22 (mutated to Gly or Lys in Arctic and Italian mutants, respectively) does not directly coordinate within the Cu(II) complex; however, there is a structural explanation for this switch in behavior. Many of the familial point mutations are situated at residue 22 and 23 and have been link with the formation of a salt-bridge to Lys28.^78^ Molecular structures of Arctic Aβ40 fibrils indicates a basic “S”-shaped topology which is much closer to the appearance of longer wild-type Aβ42.^79–81^ Indeed, we have recently shown that Arctic Aβ40 fibrils can cross-seed wild-type Aβ42 fibril formation, which suggests a very similar and compatible structure.^75^ Similarly, Aβ40 Osaka familial mutant (E22Δ) has an “S”-shaped arrangement similar to wild-type Aβ42.^82^

Furthermore, residues 22 and 23 are a “hot-spot” for mutations associated with early on-set AD, consequently there is much interest in the turn formed at these residues, this has been explored by incorporation of a D-chiral center^83^ or a lactam ring.^84^

As an aside from the Cu(II) loaded studies, we have also compared the twist periodicity and fibril width for the various isoforms studied including Arctic and Italian mutants, Figure 8I,J with that of wild-type Aβ, Figure 1D,E and also N- and C-blocked Aβ42 shown in Figure 7K,L, in the absence of Cu(II). It is notable that the shorter Aβ40 isoforms for wild-type and familial mutants tend to have a longer twist periodicity than the various Aβ42 fibrils, both wild-type and mutant. Related studies of fibril twist morphology for familial mutants have been reported.^85,86^

## Conclusion

The differential Cu(II) induced fibril/oligomer formation for wild-type Aβ40 compared to the other Aβ isoforms has ramifications for Aβ toxicity in vivo. It has long been assumed that the heightened toxicity for wild-type Aβ42 and the familiar mutants is linked with elevated amyloidogenicity, relative to the nonpathogenic wild-type Aβ40. Here, we show an even more marked contrast in behavior, which could be linked with Cu(II) trapping Aβ as oligomers for Aβ42 and the familiar mutants (such as Arctic Aβ40 and Italian Aβ40 mutants). In vivo, familial mutants of Aβ40 isoforms out-number Aβ42 in a 9:1 ratio.^87,88^ Thus, for those with the Arctic or Italian mutation, physiological Cu(II) will trap the much more abundant Aβ40 (Arctic) or Aβ40 (Italian) as toxic oligomers. This will increase the level of Cu(II) trapped toxic oligomers 9-fold, relative to wild-type Aβ. This might explain why these particular mutations cause early onset AD. Our studies suggest Cu(II) chelation could conceivably be a therapeutic approach,^33^ by reducing the amount of toxic oligomers present in vivo.

## Methods

### Aβ Peptides

All Aβ peptides were purchased from EZBiolab Inc. in a lyophilized form. Aβ peptides were synthesized using solid-phase F-moc (*N*-(9-fluorenyl)methoxycarbonyl) chemistry, and were purified with reverse-phase high performance liquid chromatography. The sequence was confirmed by mass spectrometry. The following amino acid sequences were generated:

Wild-type Aβ40 DAEFR HDSGY EVHHQ KLVFF AEDVG SNKGA IIGLM VGGVV.

Wild-type Aβ42 DAEFR HDSGY EVHHQ KLVFF AEDVG SNKGA IIGLM VGGVV IA.

Arctic Aβ40: DAEFR HDSGY EVHHQ KLVFF AGDVG SNKGA IIGLM VGGVV.

Arctic Aβ42: DAEFR HDSGY EVHHQ KLVFF AGDVG SNKGA IIGLM VGGVV IA.

Arctic Aβ42: DAEFR HDSGY EVHHQ KLVFF AGDVG SNKGA IIGLM VGGVV IA.

Arctic Aβ42: DAEFR HDSGY EVHHQ KLVFF AGDVG SNKGA IIGLM VGGVV IA.

Italian Aβ40: DAEFR HDSGY EVHHQ KLVFF AKDVG SNKGA IIGLM VGGVV.

Italian Aβ42: DAEFR HDSGY EVHHQ KLVFF AKDVG SNKGA IIGLM VGGVV IA.

N-terminally acylated: Aβ42.

D(acylated)AEFR HDSGY EVHHQ KLVFF AEDVG SNKGA IIGLM VGGVV IA.

C-terminally amidated: Aβ42.

DAEFR HDSGY EVHHQ KLVFF AEDVG SNKGA IIGLM VGGVV IA(amidated).

Unless otherwise stated, all other chemicals were purchased from Sigma-Aldrich.

### Monomeric Aβ by SEC

Aβ peptides were solubilized in ultrahigh quality water to 0.7 mg mL^–1^ and adjusting to pH 10 with NaOH and left at 4 °C for 30 min. Then, samples were centrifuged for 10 min at 20,000*g* at 4 °C, to remove any high molecular weight aggregates. In order to generate a seed-free preparation, size-exclusion chromatography (SEC) was used to remove any remaining nucleating and oligomeric aggregates; see Figure S13. Monomeric Aβ was isolated by AKTA FPLC with a Superdex 75 10/300 GL column (volume = 24 mL; GE Healthcare) with a flow rate of 0.5 mL·min^–1^ at 4 °C. The column was pre-equilibrated with 30 mM 4-(2-hydroxyethyl)-1-piperazineethanesulfonic acid (HEPES), 160 mM NaCl buffer at pH 7.4. The Aβ peptide concentrations were determined using tyrosine 10 absorption at 280 nm, ε_280_ = 1280 cm^–1^ mol^–1^. Negative-stain electron microscopy and thioflavin T fluorescence assay confirmed that SEC-purified Aβ peptides were seed-free. Monomeric Aβ were used directly after SEC elution.

### SEC Fibril Growth Assay

The kinetics of amyloid fibril formation were monitored with thioflavin T (ThT), a dye which widely employed for monitoring amyloid fibril formation.^89^ 10 μM monomeric Aβ peptides and 20 μM ThT were placed in a 96-well plate in 30 mM HEPES and 160 mM NaCl buffer at pH 7.4 at 30 °C. The well plate remained quiescent. ThT fluorescence was recorded using a FLUOstar Omega microplate reader (BMG Labtech, Aylesbury, UK), with excitation filters at 440 nm and emission filters at 490 nm. Kinetic assays in the presence of Cu^2+^ were performed under the same conditions. In the seeded aggregation assay, seeds (Aβ fibrils) 5%; 0.5 μM Aβ monomer equivalent were obtained by incubating 10 μM Aβ peptides in 30 mM HEPES and 160 mM NaCl buffer at 30 °C for 5 days. The formation of Aβ fibrils was confirmed by the ThT fluorescent assay and TEM imaging.

### Fitting Fibril Growth Curves

The empirical kinetic values for the time at which lag-times (*t*_lag_, time required for the ThT fluorescence to reach 10% of the maximum value), half maximal fluorescence is reached (*t*_50_) and transition time from 10 to 90% of the maximum value (*t*_growth_) were extracted from the data by fitting the fibril growth curve to the following equation.^90^
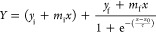
where *Y* represents the ThT fluorescence intensity and *x* represents the time. *x*_0_ is the time at which half maximal ThT fluorescence is reached, referred to as *t*_50_. The lag-time (*t*_lag_) is taken from *t*_lag_ = *x*_0_ – 2τ. The initial and final fluorescence signals is represented by *y*_i_ and *y*_f_, respectively.^90^

### Analysis of Aβ Aggregation Kinetics

AmyloFit online platform was used for the global kinetic analysis of amyloid formation.^67^ The Aβ aggregation traces are described by the following integrated rate law, based on Michaelis–Menten-like kinetics
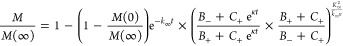
where the additional coefficients are functions of κ and λ
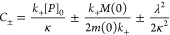

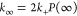

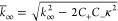

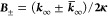
which are two combinations of the microscopic rate constants of
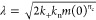

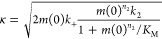
where *m*(0) is the initial monomer concentration; *M*(0), *P*(0), *M*(∞) and *P*(∞) are the fibril mass concentration and fibril number concentration in the initial and at equilibrium of the aggregation, respectively. The microscopic rate constants for primary nucleation (*k*_n_), secondary nucleation (*k*_2_), and the elongation rate (*k*_+_). *K*_M_ is the saturation constant for the secondary nucleation. The exponents *n*_c_ and *n*_2_ are the reaction orders for primary and secondary nucleation, respectively.

Aβ fibril assembly was fitted to a secondary nucleation model.^91^ The experimental macro kinetic traces were globally fitting to the integrated rate law over the range of Cu^2+^ concentrations. The microscopic rate constants *k*_n_, *k*_+_, and *k*_2_ were fitted to the Aβ fibril growth curves in the absence of Cu^2+^. The other kinetic traces at increasing Cu^2+^ concentrations, were then fitted in three scenarios in which only one of the rate constants were permitted to vary, while the other two remain as single (globally fitted) constants. With the initial monomer concentration fixed as a global constant (10 μM), *n*_c_ and *n*_2_ were set as global constant of 2 so as not overfit the data. This approach has been used to study how increasing concentrations of an inhibitor of fibril formation effect individual microrate constants.^92–94^

### Transmission Electron Microscopy

Aβ fibril samples were generated with the same protocol for Aβ fibril growth assay but without ThT addition. 5 μL aliquot of sample were added onto glow discharged 300 mesh carbon-coated copper grids (Agar Scientific Ltd.) using the droplet method, with ddH_2_O washes before and after addition of stain. Glow discharge was carried out by the EasiGlow glow discharge cleaning system (Pelco inc, USA). 5 μL of uranyl acetate (2% w/v) was used to negatively stain the samples, then blotted and rinsed after 10 s at room temperature. Imaging was carried out on a JEOL JEM-1230 electron microscope (JEOL, Ltd., Japan) at 80,000 magnifications, operated at 120 kV, paired with a 2k Morada CCD camera and corresponding microscope image analysis software (Olympus Europa, UK). Node-to-node fibril distance was measured by ImageJ software.

### Cellular Ca(II) Influx

Fluo3-AM-loaded HEK293T cells: HEK293T cells were incubated at 37 °C, in a 5% CO_2_ incubator, in Dulbecco’s modified Eagle’s medium (DMEM, purchased from Thermo Fisher) supplemented with 10% fetal bovine serum and penicillin–streptomycin (0.2 mg/mL). Cells were plated into a 12-well plate, 1 mL each well, and incubated overnight, cells typically gained ca. 70% confluence. Next medium was replaced with fresh DMEM which was supplemented with 5 μM Fluo3-AM (Abcam). To enable the cellular uptake of the Ca^2+^ sensitive fluorescent dye, plates were left in an incubator for a further 30 min. Excess extracellular Fluo3-AM was removed by two washes of 400 μL of DMEM Eagles cell medium in each well. Cells were then incubated for a further 20 min at 37 °C to allow de-esterification of intracellular Fluo-3 to occur, which activates Ca^2+^ dependent fluorescence. Finally, the DMEM was replaced with an aqueous buffer containing CaCl_2_ (1.8 mM); NaCl (120 mM); CsCl (10 mM); HEPES (9 mM); KCl (2.2 mM); and MgCl_2_ (1.9 mM), buffered to pH 7.4. The Fluo-3 loaded cells were then ready for time-lapse fluorescence microscopy.

Ca^2+^ Fluorescence Imaging of Fluo-3: Fluorescence microscopy was performed using an inverted Leica DM IL microscope with a magnification of 10×. The bandpass filter allowed excitation at 470 nm, and emission was recorded at 520 nm. Time-lapse fluorescence images and bright-field visible light images were acquired using a charge-coupled device (CCD) camera with a temporal resolution of one image every 5 s; recordings were for 10 min.

The microscope was operated using ProgRes CapturePro 2.8.8 software, and fluorescence intensities were measured by analyzing the total field (typically ca. 500 individual cells), using the time series analyzer V3 plugin and ImageJ. Changes in Fluo-3 fluorescence signals are presented as (*F*/*F*_0_) – 1, where (*F*) is the observed fluorescence and (*F*_0_) is the background fluorescence at a time point just before the addition of Aβ. Typically, each condition was recorded using three separate wells, with independent repeats.

The impact of five Aβ42 preparations were studied, including: monomers (SEC purified); oligomers (from the end of the lag-phase); fibrils (from the plateau-phase); Cu(II) trapped oligomers; and Cu(II) disassembled fibrils. Stock solutions of 30 μM Aβ was added to HEK293 cells within 400 μL of cell medium to produce a final Aβ (monomer equivalent) concentration of 5 μM.

## Supporting Information Available

Kinetic data, TEM images of various Aβ assemblies with and without the presence of Cu^2+^, ThT monitor fibril formation kinetics, and chromatograms of SEC-purified Aβ monomers (PDF)

## Abbreviations

AD: Alzheimer’s disease
Aβ: amyloid-beta
ThT: thioflavin T
TEM: transmission electron microscopy
SEC: size exclusion chromatography
