# Supplementary material for: Copper(II) Can Kinetically Trap Arctic and Italian Amyloid-β40 as Toxic Oligomers, Mimicking Cu(II) Binding to Wild-Type Amyloid-β42: Implications for Familial Alzheimer’s Disease
Source: JACS Au. 2024 Feb 6;4(2):578–91. doi: 10.1021/jacsau.3c00687 (PMC10900208; doi:10.1021/jacsau.3c00687)
Supplement: Supplementary file 1 — au3c00687_si_001.pdf [file au3c00687_si_001.pdf]

Supplementary Information

Copper(II) can kinetically trap Arctic and Italian amyloid-β_{40} as toxic oligomers, mimicking Cu(II) binding to wild-type amyloid-β_{42}: implications for familial Alzheimer's disease

Yao Tian, Qi Shang, Ruina Liang, and John H. Viles*

Corresponding Author

John Viles – School of Biological and Behavioral Sciences, Queen Mary University of London, London, E1 4NS, United Kingdom. Email: j.viles@qmul.ac.uk

Authors

Yao Tian – School of Biological and Behavioral Sciences, Queen Mary University of London, London, E1 4NS, United Kingdom

Qi Shang – School of Biological and Behavioral Sciences, Queen Mary University of London, London, E1 4NS, United Kingdom

Ruina Liang – School of Biological and Behavioral Sciences, Queen Mary University of London, London, E1 4NS, United Kingdom

Table S1. t_{50}, t_{growth} and ThT intensity of Aβ in the absence and presence of 0.5 and 1.0 molar equivalent of Cu(II)

	t_{50}				t_{growth}				ThT intensity			
	+0 Cu	+0.1 Cu	+0.5 Cu	+1.0 Cu	+0 Cu	+0.1 Cu	+0.5 Cu	+1.0 Cu	+0 Cu	+0.1 Cu	+0.5 Cu	+1.0 Cu
Aβ40	56.0 h	46.7 h	31.5 h	24.9 h	9.2 h	7.1 h	8.2 h	7.3 h	34569	35524	33876	35998
Aβ42	35.6 h	40.7 h	49.8 h	67.3 h	8.6 h	9.3 h	9.0 h	9.6 h	43923	35833	13263	5019
C-amidated Aβ42	0.93 h	0.97 h	1.05 h	1.07 h	9.2 h	7.1 h	8.2 h	7.3 h	32840	30659	25677	20520
N-truncated Aβ42	58 h	69.5 h	–	–	25.1 h	26.6 h	–	–	39756	31445	7833	2715
Arctic Aβ40	11.5 h	14.9 h	–	–	6.0 h	6.7 h	–	–	40972	34826	9407	3917
Arctic Aβ42	8.1 h	10.7 h	12.7 h	–	4.2 h	4.6 h	4.4 h	–	39190	34616	14820	9895
Italian Aβ40	43.7 h	52.1 h	–	–	11.8 h	13.0 h	–	–	42546	35816	11083	7187
Italian Aβ42	58 h	65.3 h	–	–	34. h	36.5 h	–	–	41493	34444	17968	4236

Gray boxes indicate t_{50} and ThT intensity decrease from 0 to 1.0 molar equivalent Cu(II). Red boxes indicate t_{50} increase from 0 to 1.0 molar equivalent Cu(II).

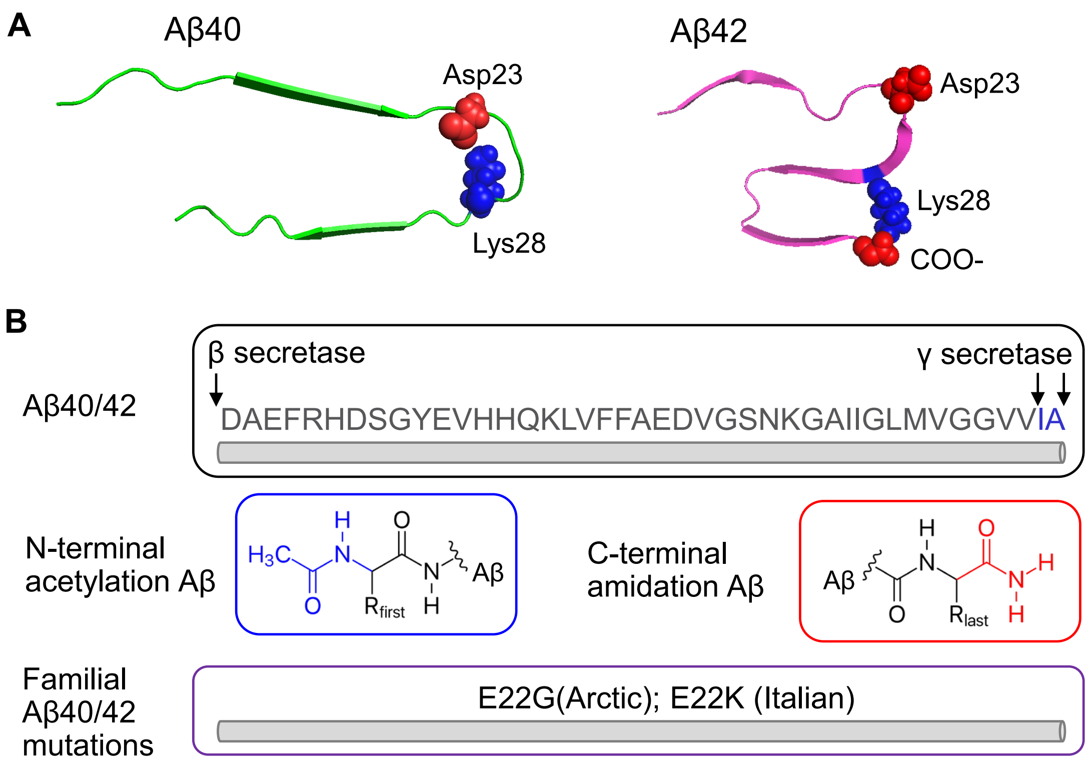

Figure S1. Structure and sequence of wild-type Aβ and its variants. (A) Aβ40 and Aβ42 fibril structure (PDB: 2LMO and 2MXU). Fibril topology of Aβ40 'U' shaped structure and Aβ42 'S' shaped structure. Columbic interaction between Asp23, Lys28 and C-terminus are highlighted. (B) Amino acid sequence of Aβ and its isoforms studied in this work.

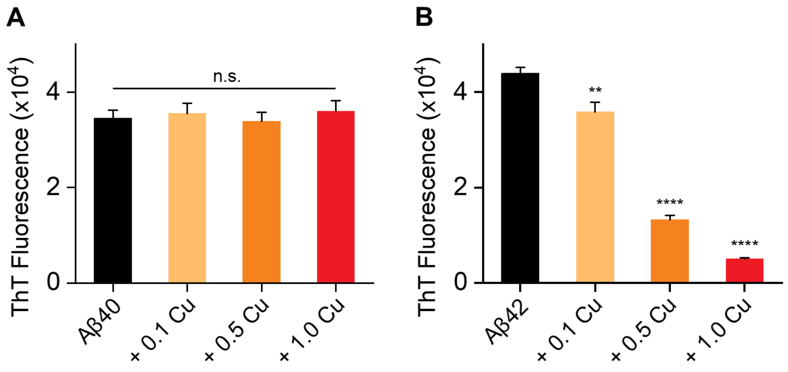

Figure S2. ThT fluorescence intensity versus Cu^{2+}. Aβ40 (A) and Aβ42 (B). Derived from data in Figure 1. Error bars are standard error of the mean (SEM) from four replicates. One-way ANOVA test, $**P \leq 0.01$, $****P \leq 0.0001$.

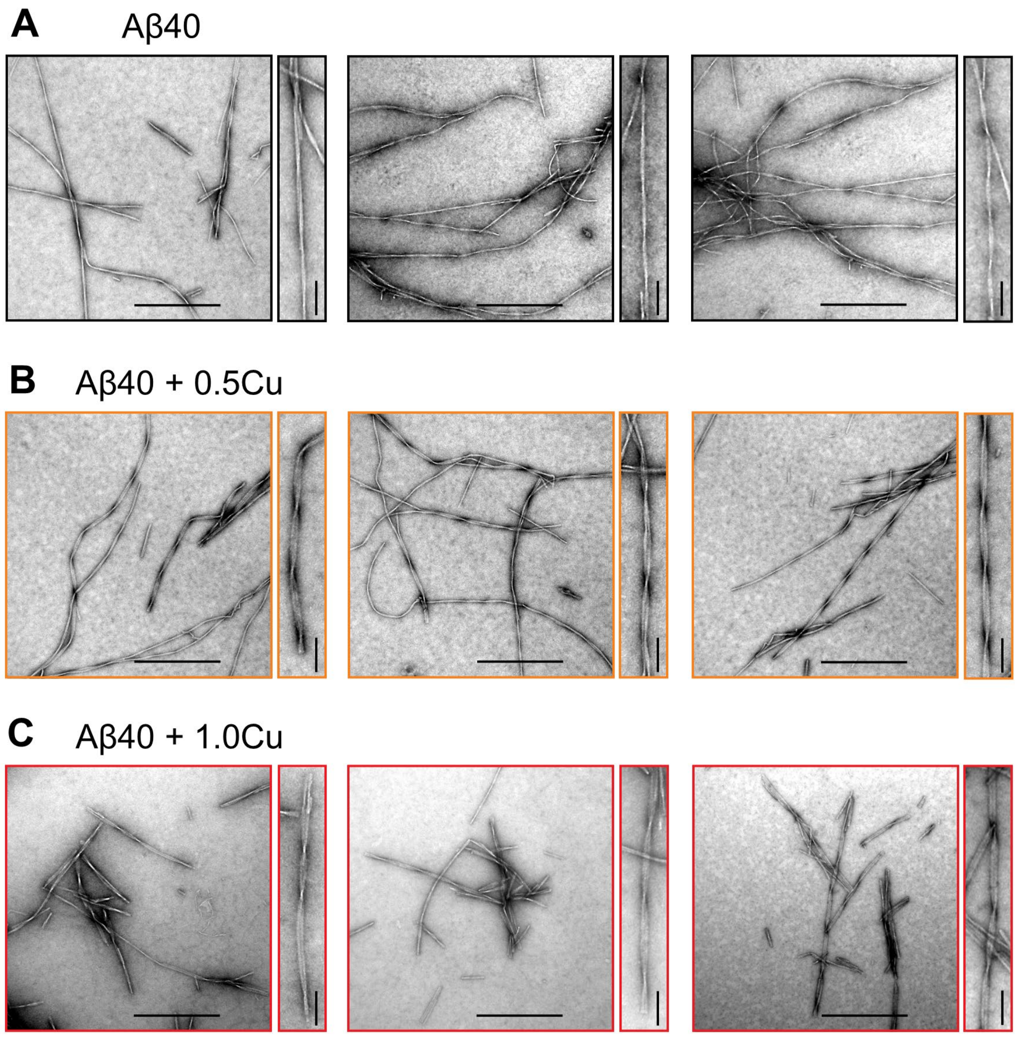

Figure S3. TEM images of Aβ40 in the absence (A) and presence of 0.5 (B) and 1.0 (C) molar equivalents of Cu(II). Scale bars: 500 nm; inset 100 nm.

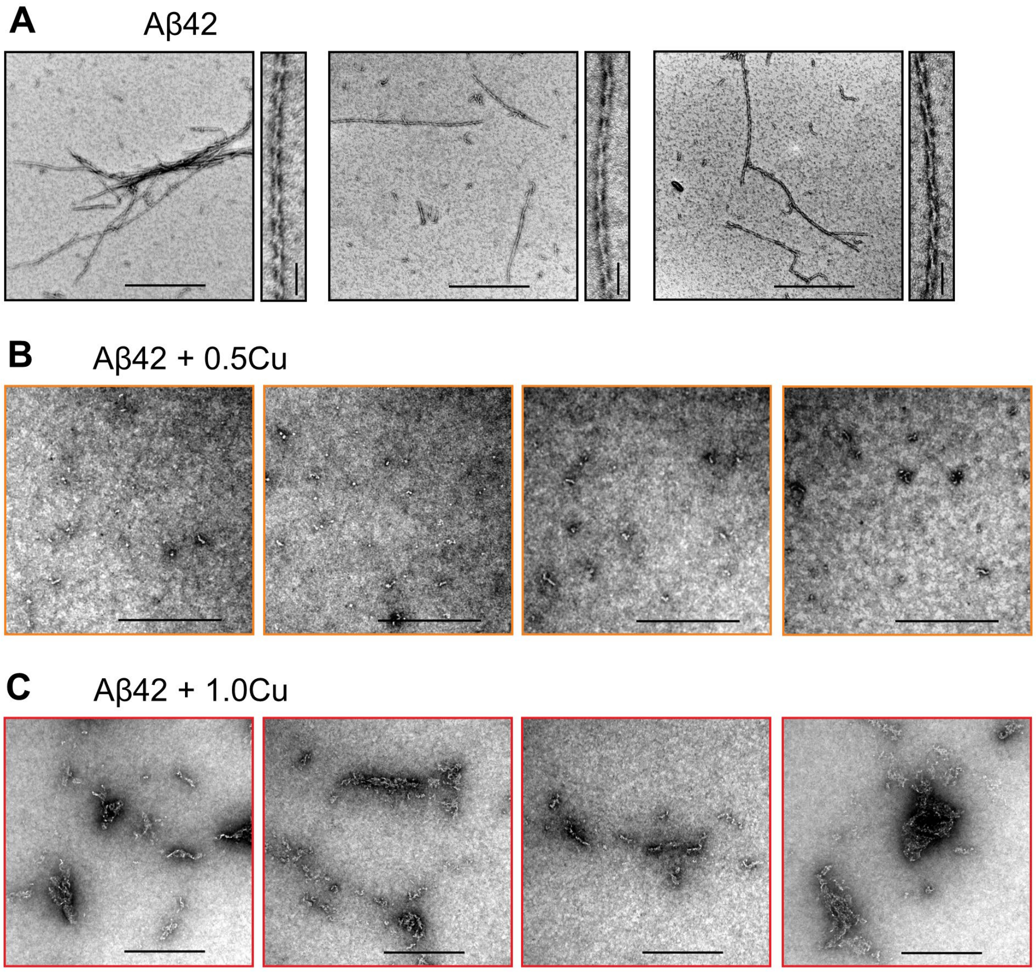

Figure S4. TEM images of Aβ42 in the absence (A) and presence of 0.5 (B) and 1.0 (C) molar equivalents of Cu^{2+}. Scale bars: 500 nm; inset 50 nm.

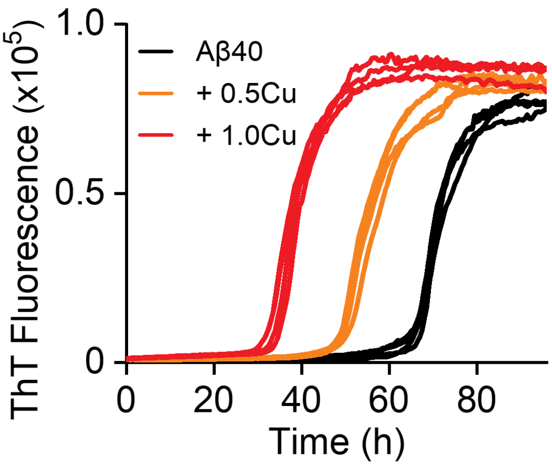

Figure S5. Cu(II) also promotes Aβ40 fibril formation in sodium phosphate buffer. Kinetics profiles of 20 μM Aβ40 in the absence and presence of 0.0, 0.5 and 1.0 molar equivalents of Cu(II), from black line to red line, respectively. Preparations were incubated with 20 μM ThT in 20 mM sodium phosphate buffer, pH 7.2, at 30 °C.

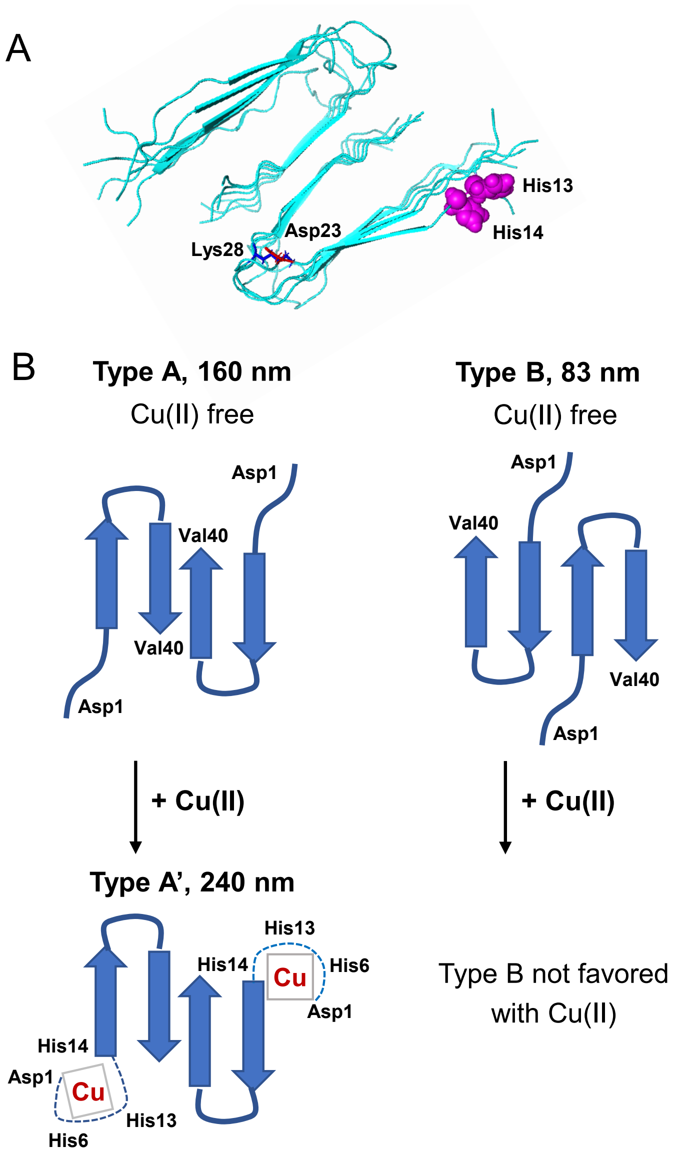

Figure S6. (A) Structure of Aβ40 fibrils with Cu(II) binding histidine sidechains highlighted (PDB=2LMO). (B) Cartoon showing how Cu(II) could impact the packing of protofibrils and so affect the morphology of fibrils. Mean node-to-node fibril twist is indicated with and without the presence of Cu(II).

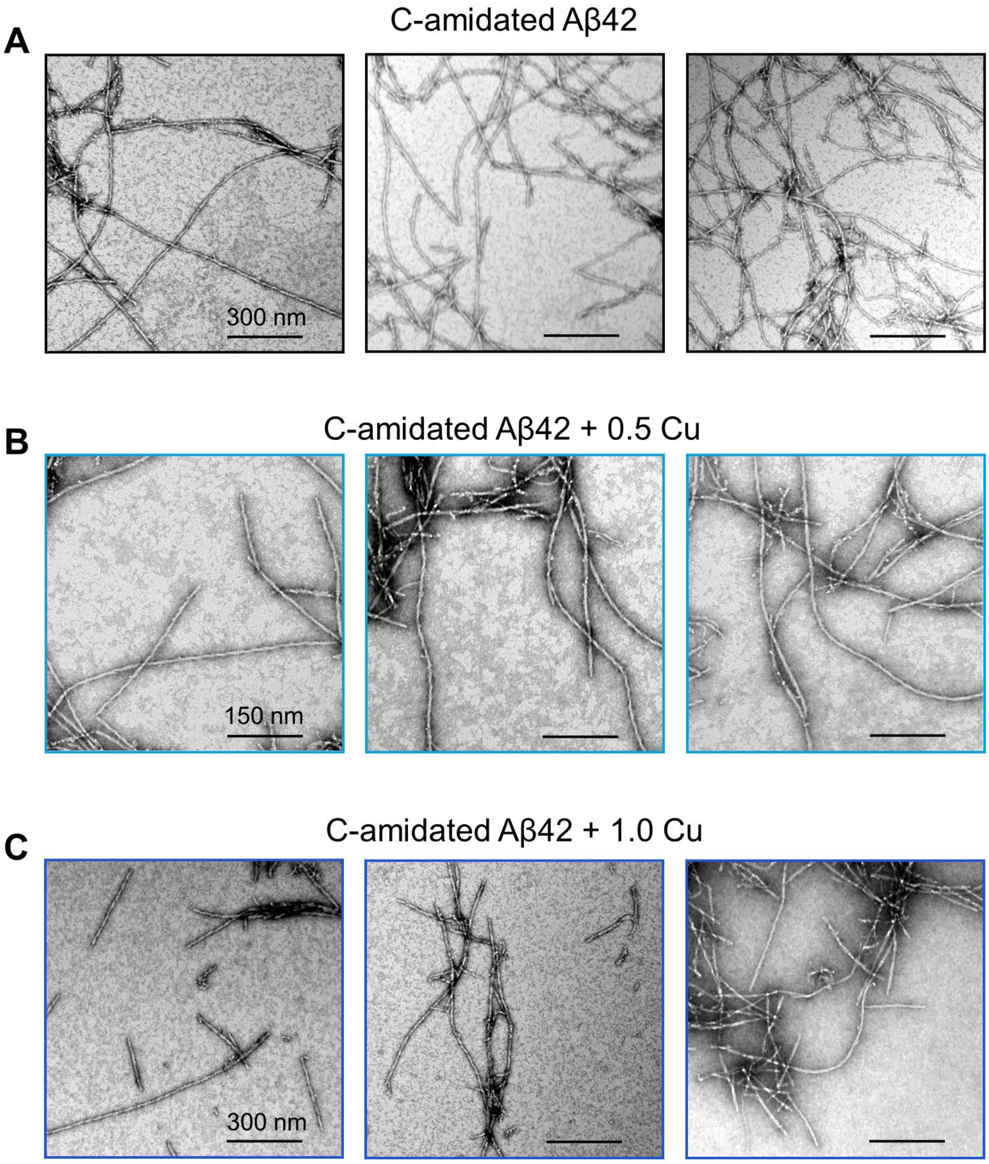

Figure S7. TEM images of C-amidated Aβ42 in the absence (A) and presence of 0.5 (B) and 1.0 (C) molar equivalents of Cu^{2+}. Scale bars 300 nm. Unlike wild-type Aβ42, Fibrils dominate images with or without the presence of Cu(II).

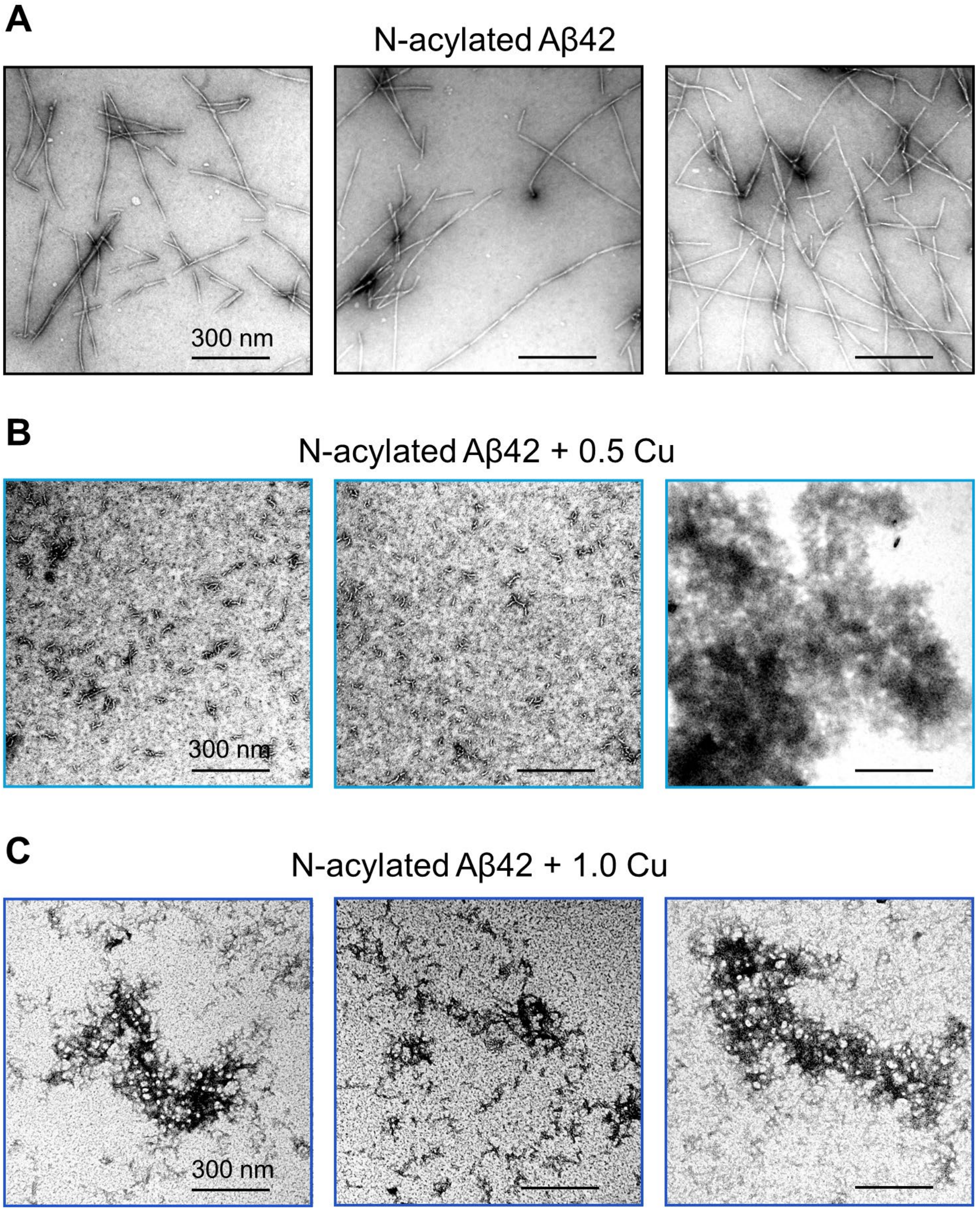

Figure S8. TEM images of N-acylated Aβ42 in the absence (A) and presence of 0.5 (B) and 1.0 (C) molar equivalents of Cu^{2+}. Scale bars 300 nm. Like wild-type Aβ42, Cu(II) traps N-acylated Aβ42 as protofibrils.

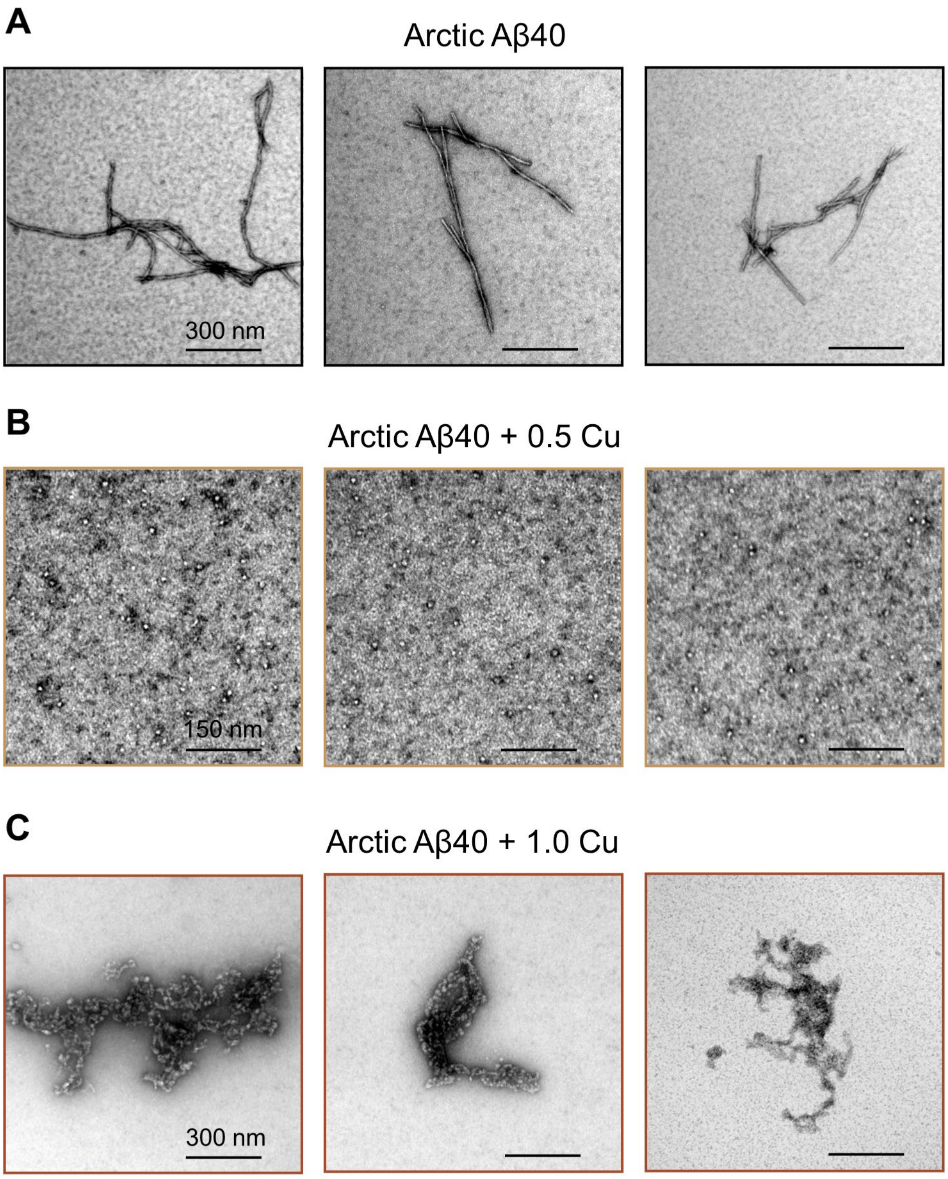

Figure S9. Cu(II) traps Arctic Aβ40 as protofibrils. TEM images of Arctic Aβ40 in the absence (A) and presence of 0.5 (B) and 1.0 (C) molar equivalents of Cu^{2+}. Scale bars 300 nm for (A) and (C), 150 nm for (B).

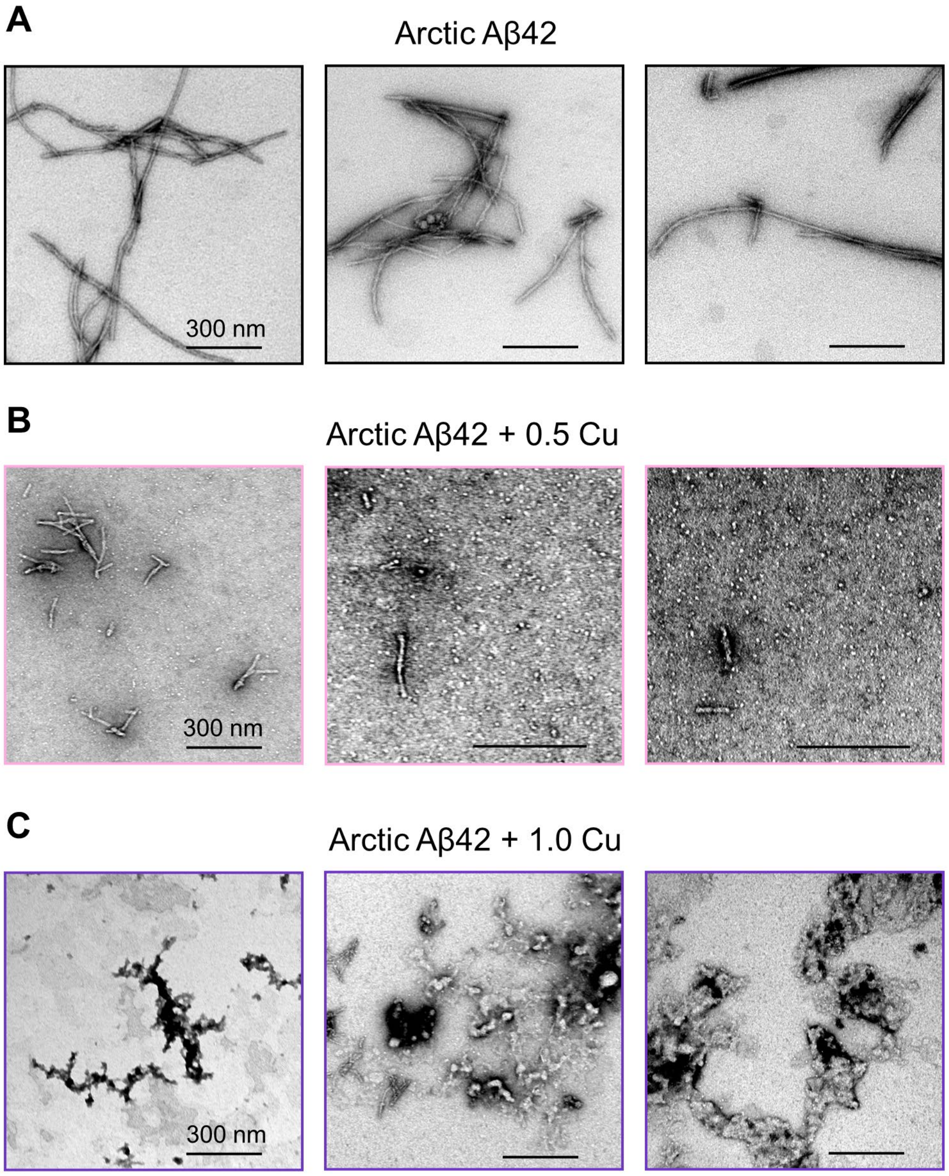

Figure S10. Cu(II) traps Arctic Aβ42 as protofibrils. TEM images of Arctic Aβ42 in the absence (A) and presence of 0.5 (B) and 1.0 (C) molar equivalents of Cu(II), Scale bars: 300 nm. At one molar equivalent of Cu(II) amorphous aggregates are observed.

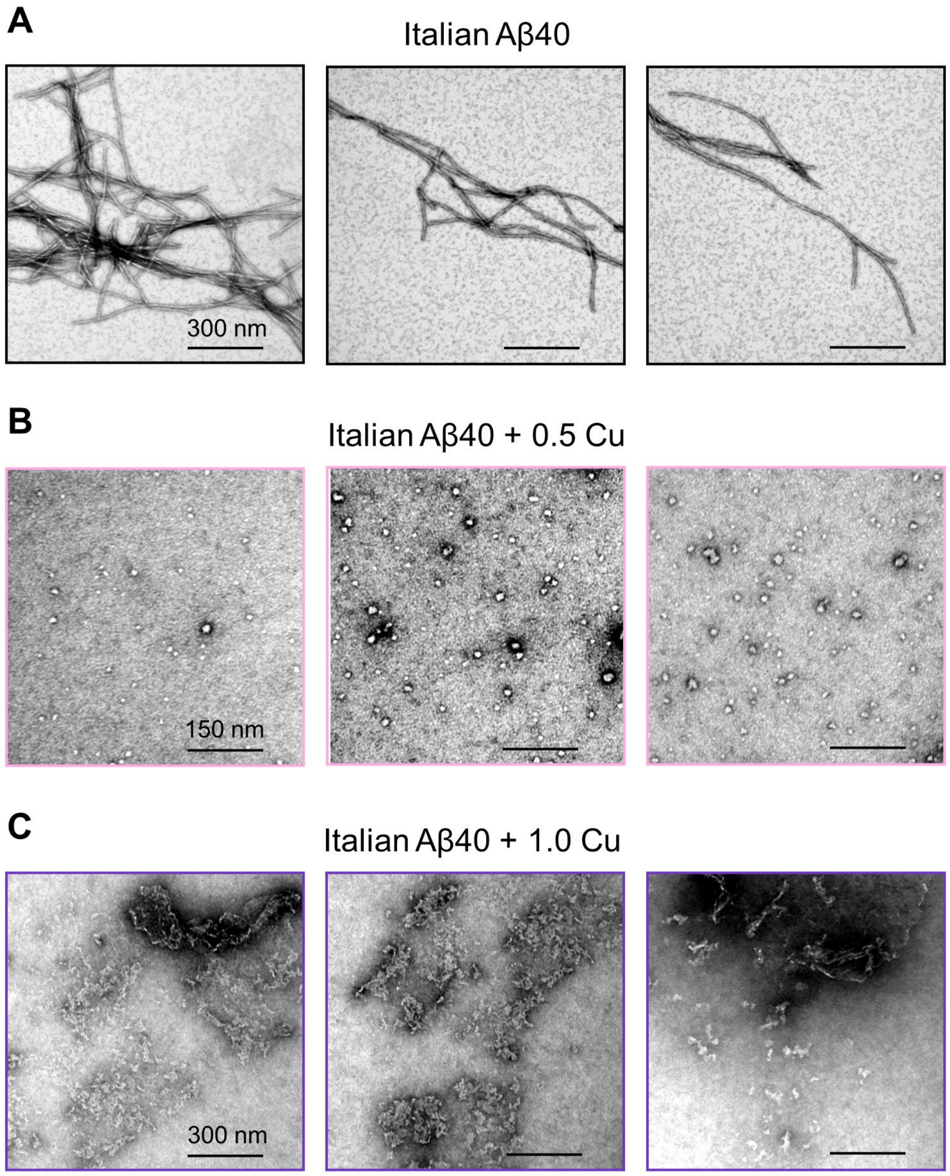

Figure S11. Cu(II) traps Italian Aβ40 as protofibrils. TEM images of Italian Aβ40 in the absence (A) and presence of 0.5 (B) and 1.0 (C) molar equivalents of Cu^{2+}. Scale bars 300 nm for (A) and (C), 150 nm for (B).

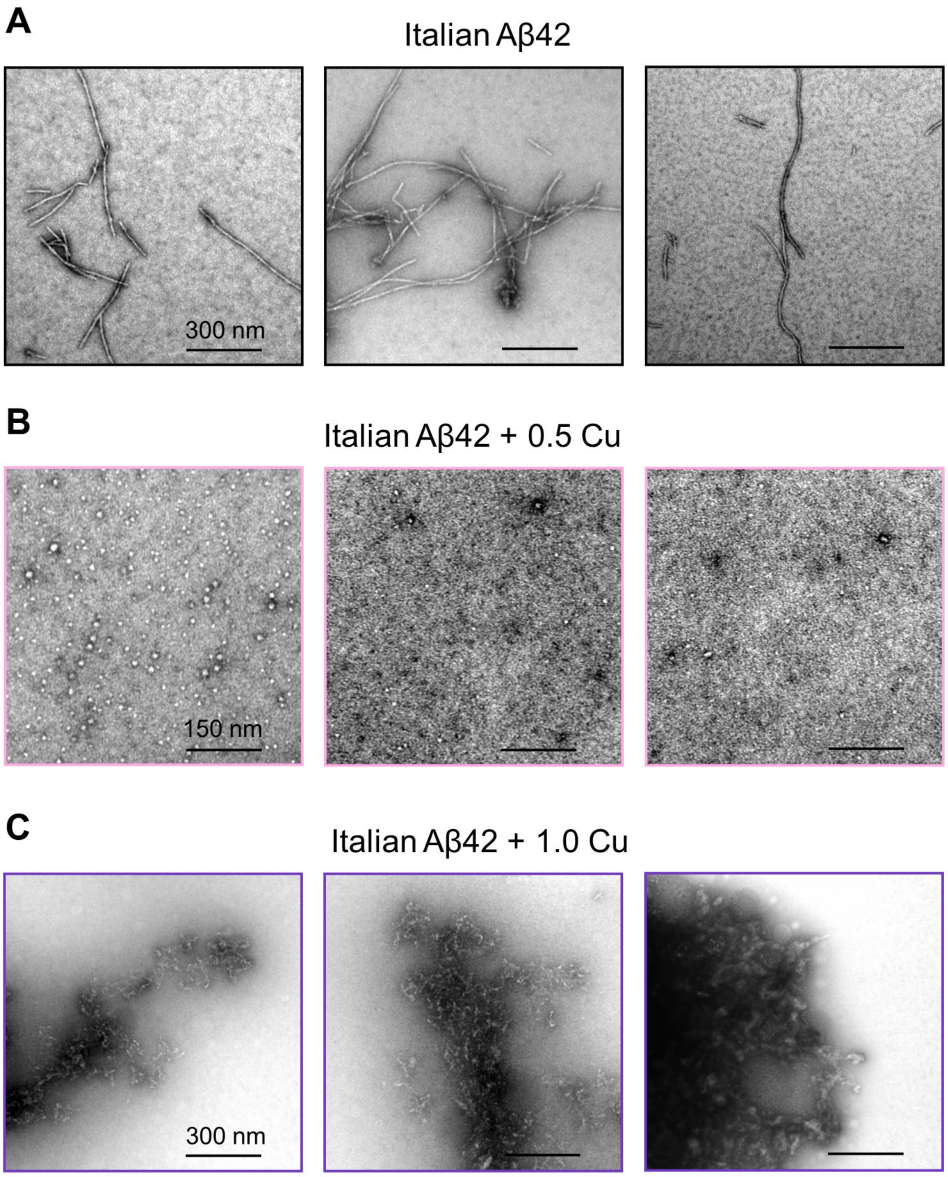

Figure S12. Cu(II) traps Italian Aβ42 as protofibrils. TEM images of Italian Aβ42 in the absence (A) and presence of 0.5 (B) and 1.0 (C) molar equivalents of Cu^{2+}. Scale bars 300 nm for (A) and (C), 150 nm for (B).

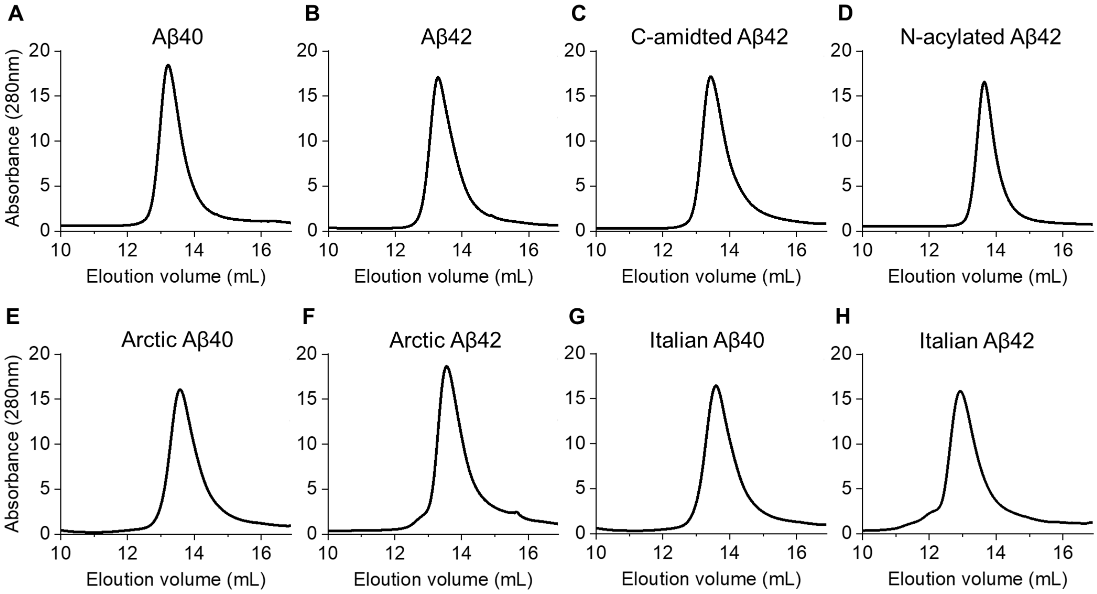

Figure S13. Isolation of Aβ monomer. SEC elution profile (280 nm) indicates the elution of a single monomeric fraction of (A) Aβ40, (B) Aβ42, (C) C-amidated Aβ42, (D) N-acylated Aβ42, (E) Arctic Aβ40, (F) Arctic Aβ42, (G) Italian Aβ40 and (H) Italian Aβ42. The Aβ monomeric samples were taken directly from the SEC column elution.
